# Igfbp2 Downregulation in PVT‐CeA Glutamatergic Circuits Drives Neonatal Anesthesia‐Induced Fear Memory Deficits

**DOI:** 10.1002/advs.202505025

**Published:** 2025-08-13

**Authors:** Weiming Zhao, Ke Peng, Baojian Zhao, Xiaowen Meng, Gang Wang, Hanbing Xu, Li Deng, Xisheng Shan, Yichan Wang, Qiya Xu, Yanan Gao, Ruixia Weng, Hong Liu, Jin Tao, Huayue Liu, Shaoyong Song, Fuhai Ji

**Affiliations:** ^1^ Department of Anesthesiology First Affiliated Hospital of Soochow University Suzhou Jiangsu China; ^2^ Institute of Anesthesiology Soochow University Suzhou Jiangsu China; ^3^ Department of Anesthesiology Nanjing Stomatological Hospital Affiliated Hospital of Medical School Nanjing University Nanjing Jiangsu China; ^4^ Department of Gastroenterology First Affiliated Hospital of Soochow University Suzhou Jiangsu China; ^5^ Department of Anesthesiology and Pain Medicine University of California Davis Health System Sacramento CA 95817 USA; ^6^ School of Basic Medical Sciences Suzhou Medical College of Soochow University Suzhou Jiangsu China; ^7^ Ambulatory Surgery Center First Affiliated Hospital of Soochow University Suzhou Jiangsu China

**Keywords:** anesthesia, central amygdala, fear memory, Insulin‐like growth factor‐binding protein 2, paraventricular thalamus

## Abstract

Repeated neonatal general anesthesia results in long‐term cognitive dysfunction; however, the underlying mechanisms remain unclear. This study finds that repeated neonatal anesthesia impaired fear memory in adolescent mice of both sexes, along with hypoactivated glutamatergic neurons in the paraventricular thalamus (PVT). Optogenetic activation of PVT glutamatergic neurons rescued fear memory deficits in anesthesia‐treated mice, whereas optogenetic inhibition of these neurons recapitulated memory deficits in control mice. Specifically, repeated neonatal anesthesia reduced insulin‐like growth factor‐binding protein 2 (Igbp2) expression and dendritic spine density in PVT glutamatergic neurons in both males and females. Selectively manipulating PVT glutamatergic Igfbp2 mediated anesthesia‐induced fear memory deficits through modulating neuron excitability and spine density. Notably, optogenetic activation or restoring Igfbp2 expression in glutamatergic projections from the PVT to the central amygdala (CeA) blocked anesthesia‐induced memory impairment, whereas optogenetic inhibition or knocking down of Igfbp2 expression in these projections is sufficient to engender similar memory impairment in control mice. The findings demonstrate that Igfbp2 in glutamatergic neurons in the PVT afferents to the CeA mediates fear memory deficits caused by repeated neonatal anesthesia in mice of both sexes, highlighting Igfbp2 as a potential therapeutic target for repeated anesthesia‐induced cognitive impairment.

## Graphical abstract

Repeated neonatal sevoflurane exposure impairs adolescent fear memory through Igfbp2 downregulation in paraventricular thalamus‐central amygdala glutamatergic projections. Restoring Igfbp2 or activating this circuit rescues memory deficits, neuronal excitability, and spine density, identifying a precise molecular target for preventing anesthesia‐induced neurodevelopmental cognitive impairment.

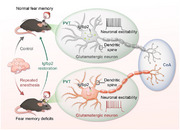

## 1 Introduction

The developing brain is particularly vulnerable to various environmental and pathophysiological factors.^[^ ^1^ ^]^ Pediatric epidemiological studies linked repeated or prolonged, but not short‐term, exposure to general anesthetics during early life to cognitive and behavioral abnormalities, including learning disabilities and attention deficit/hyperactivity disorders.^[^ ^2^, ^3^, ^4^, ^5^ ^]^ Additionally, studies in rodents and nonhuman primates have shown that prolonged or repeated general anesthesia during the neonatal period resulted in long‐term adverse effects on cognitive development and brain structures.^[^ ^6^, ^7^, ^8^, ^9^ ^]^ Consequently, the potential long‐term adverse impact of general anesthetics on the developing brain has raised significant clinical concerns, prompting the US Food and Drug Administration to issue a safety warning regarding their use in children under 3 years of age.^[^ ^10^ ^]^

Repeated neonatal anesthesia can impact the processing of cerebral cortex and various subcortical brain regions.^[^ ^11^ ^]^ Thalamus, an evolutionarily ancient structure, plays a fundamental role in cognitive function by relaying sensory and motor signals to the cerebral cortex.^[^ ^12^, ^13^, ^14^, ^15^ ^]^ Thalamic volume reduction has been recognized as an early sign of amnestic mild cognitive impairment.^[^ ^16^ ^]^ Previous studies have shown that exposure to general anesthetics induced robust apoptotic neurodegeneration in the thalamus of neonatal rodents.^[^ ^17^, ^18^ ^]^ Paraventricular thalamus (PVT) is a key component of the dorsal midline thalamus involved in regulating the states of consciousness during general anesthesia.^[^ ^19^, ^20^, ^21^, ^22^ ^]^ Moreover, PVT glutamatergic neurons in neonatal rodents are sensitive to early‐life stressors.^[^ ^23^ ^]^ Therefore, anesthetic agents act through the PVT while also may adversely affect its structure and function, potentially leading to long‐term cognitive impairment.

Central amygdala (CeA), a key component of the amygdaloid complex, modulates fear, stress, and anxiety.^[^ ^24^, ^25^, ^26^ ^]^ Polymodal and highly processed information from thalamus and cortical areas reaches CeA, highlighting its crucial role in integrating higher brain functions, especially those related to emotion and cognition. The CeA is critically involved in learning and memory, particularly in the formation and regulation of emotion‐related memories.^[^ ^24^ ^]^ A study suggested that selective inactivation of the PVT neurons afferents to CeA prevented fear expression 24 h after fear acquisition.^[^ ^27^ ^]^ Therefore, we speculated that the projections from the PVT to the CeA may play a crucial role in modulating fear memory deficits induced by repeated neonatal anesthesia.

Insulin‐like growth factor‐binding protein 2 (Igfbp2) is one of six members of the IGF‐binding protein superfamily.^[^ ^28^ ^]^ Igfbp2 expression in the central nervous system increases from the embryonic stage to adulthood and is associated with brain development, astrocyte proliferation, and neurite outgrowth.^[^ ^29^ ^]^ A recent study revealed that Igfbp2 is crucial for early cognitive development by regulating hippocampal neuronal plasticity.^[^ ^30^ ^]^ Knocking down of Igfbp2 expression in the basolateral amygdala (BLA) astrocytes impaired fear memory expression.^[^ ^31^ ^]^ Igfbp2 also offers neuroprotection to hippocampal neurons by reducing Aβ‐induced tau phosphorylation and neuronal death in Alzheimer's disease.^[^ ^32^ ^]^ However, the effect of Igfbp2 on impaired memory induced by repeated neonatal anesthesia remains unclear.

In this study, we investigated the region‐ and cell type‐specific roles of the thalamus in repeated neonatal anesthesia‐induced fear memory impairment and the underlying Igfbp2‐mediated circuit mechanisms. To model the effects of substantial cumulative general anesthesia in children, neonatal male and female mice were repeatedly exposed to sevoflurane. Using cue fear conditioning tests, thalamic fos mapping, in vivo fiber photometry, ex vivo whole‐cell recordings, optogenetics and transcriptomic sequencing, we identified a critical role of Igfbp2 within the PVT glutamatergic neurons in regulating anesthesia‐induced memory impairment. Furthermore, these changes were mediated by glutamatergic projections from the PVT to the CeA. Our findings provide a new insight into the mechanisms of anesthesia‐induced cognitive impairment and highlight Igfbp2 as a potential therapeutic target.

## 2 Results

### 2.1 Repeated Neonatal Anesthesia Impaired Fear Memory in Adolescent Mice

**Figure 1:**
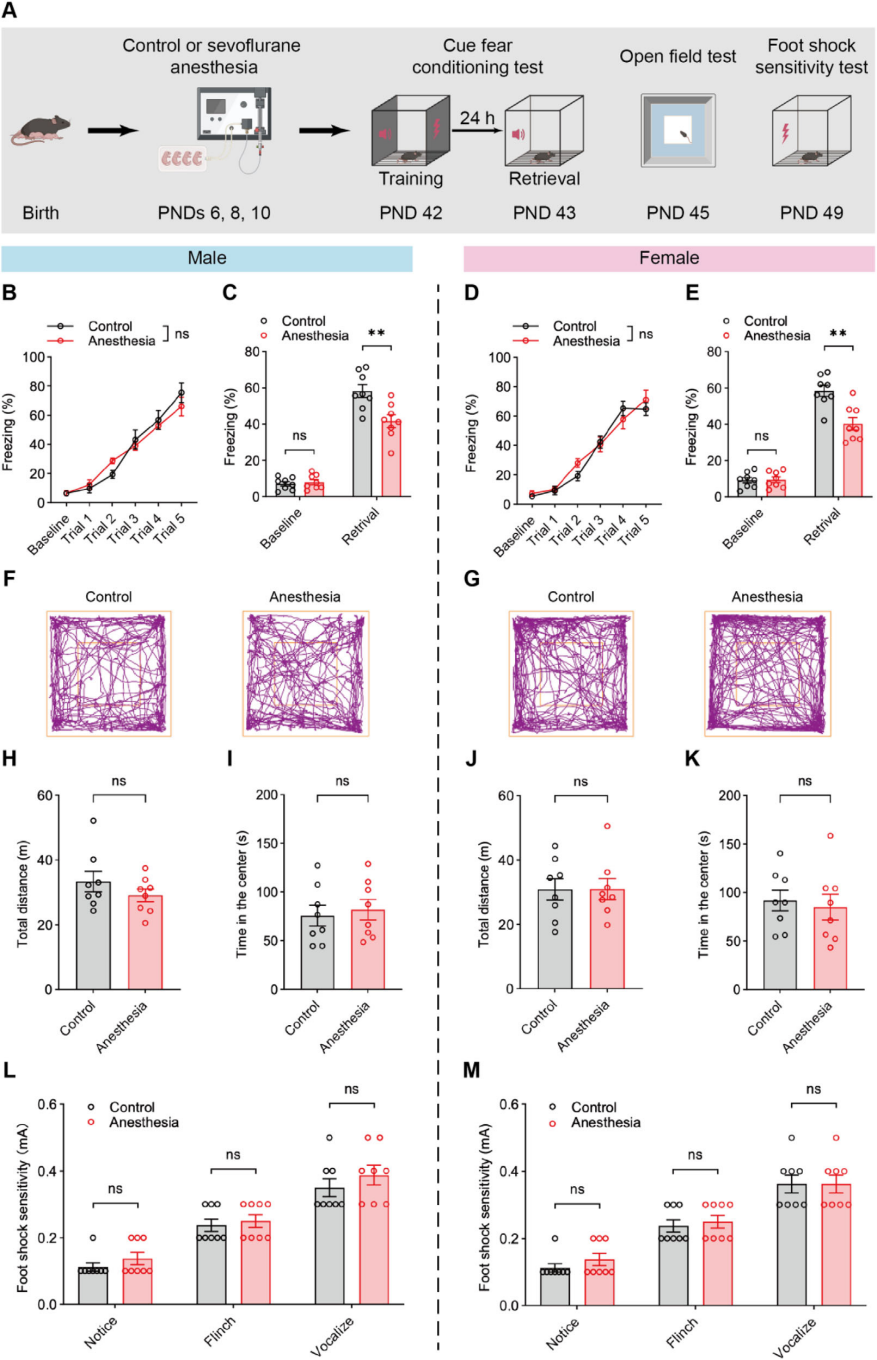
Repeated neonatal anesthesia induced fear memory deficits in both male and female adolescent mice. A) Schematic of mice undergoing repeated neonatal anesthesia and subsequent behavioral tests. B,D) No significant difference in training performance between control and anesthesia groups in males (B) and females (D). C,E) Anesthesia group showed impaired fear memory retrieval compared to the control group in males (C) and females (E), while baseline freezing levels showed no between‐group difference in either sex. F,G) Representative trajectory plots of males (F) and females (G) in open field test. H,J) Total distance traveled in the open field arena showed no significant difference between control and anesthesia groups in males (H) and females (J). I,K) Time spent in the center region of the open field arena also showed no between‐group difference in males (I) and females (K). L,M) No significant difference in foot shock sensitivity between control and anesthesia groups in males (L) and females (M). Analyzed by two‐way repeated‐measure ANOVA in B and D, and unpaired t test in C, E, H, I, J, K, L and M; n = 8 mice per group; **P < 0.01, ns: not significance. Data are presented as means ± SEM.

To investigate the long‐term effects of repeated general anesthesia on learning and memory, neonatal mice were subjected to sevoflurane anesthesia for 2 h per day on postnatal days (PNDs) 6, 8, and 10 (**Figure** **1**A).^[^ ^33^ ^]^ Given our interest in the role of the thalamus in learning and memory related to anesthesia‐induced neurodevelopmental toxicity, we utilized the cued fear conditioning test, which is sensitive to hippocampus‐independent learning and memory.^[^ ^34^ ^]^ On PNDs 42‐43, mice underwent this paradigm to assess their learning and memory capabilities.

During the training phase, both male and female anesthesia‐treated mice exhibited freezing behavior comparable to control mice (Figure 1B,D), indicating that their fear learning ability was intact. However, during memory retrieval, the anesthesia group exhibited significant fear memory deficits, with a reduced percentage of freezing time compared to the control group in both males and females (Figure 1C,E). No significant sex differences were observed in the extent of memory impairment (Figure S1, Supporting Information). These results indicated that repeated neonatal anesthesia impaired fear memory in adolescent mice of both sexes.

Further investigations confirmed that repeated neonatal anesthesia did not significantly impact locomotor activity. The open field test showed no between‐group difference in the total distance traveled in either sex (Figure 1H,J). The anesthesia‐treated mice did not exhibit anxiety‐like behavior, as demonstrated by their comparable time spent in the center of the open field arena (Figure 1I,K). Similarly, the elevated plus maze test showed no significant difference in open‐arm time between groups in either sex, further supporting that repeated anesthesia did not induce anxiety‐like behavior (Figure S2, Supporting Information). Additionally, the foot shock thresholds were also comparable between groups (Figure 1L,M), suggesting that the observed impaired memory were not due to altered sensitivity to stimuli.

### 2.2 Repeated Neonatal Anesthesia Inhibited PVT Glutamatergic Neuron Activity in Adolescent Mice

**Figure 2:**
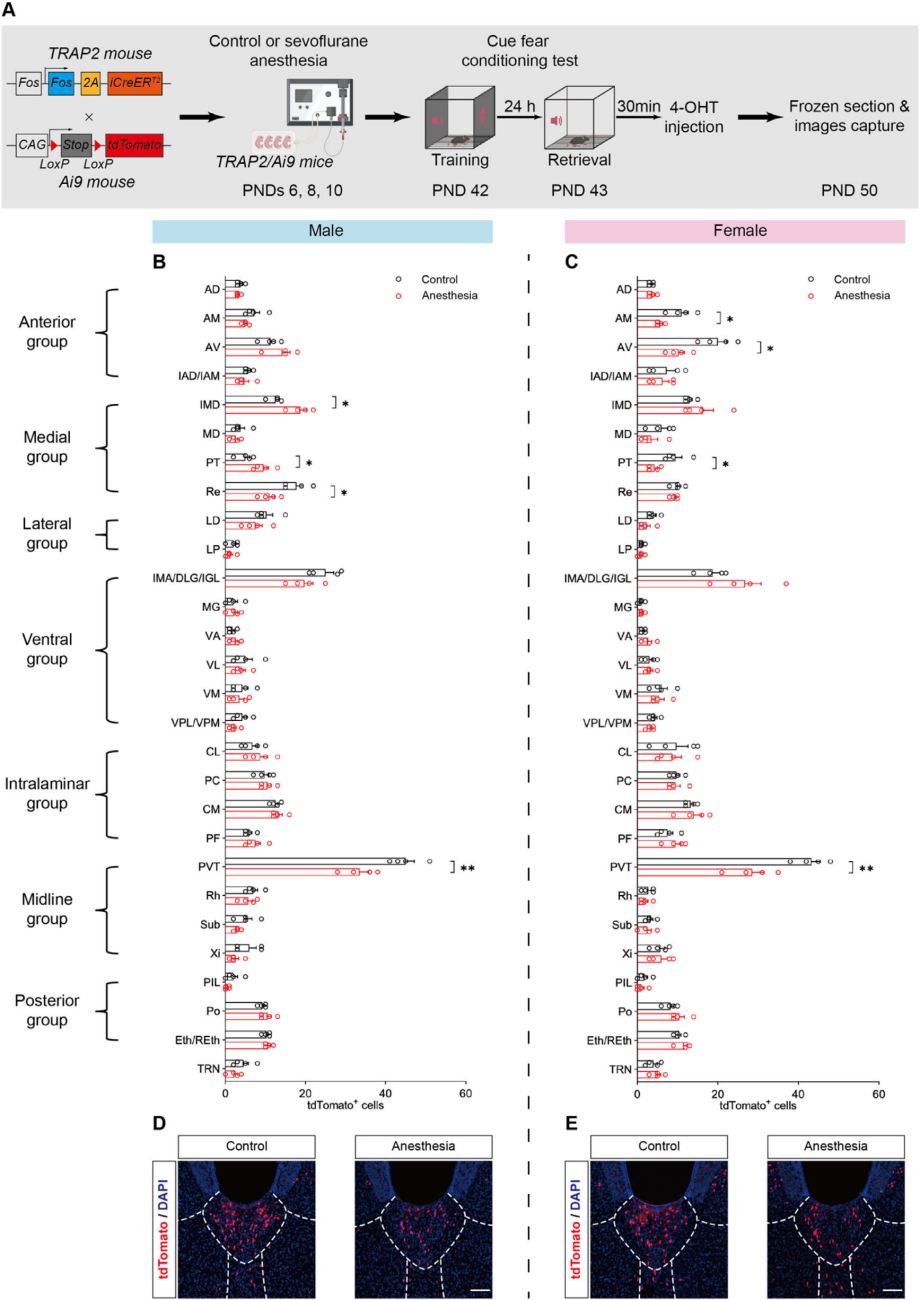
Repeated neonatal anesthesia impaired PVT activity following fear memory retrieval in both male and female mice. A) Schematic of labeling thalamic neurons related to fear memory retrieval with tdTomato in *TRAP2/Ai9* mice. B,C) Histograms summarize the tdTomato^+^ expression levels in thalamus following fear memory retrieval of males (B) and females (C); n = 4 brain sections from 4 mice per group. D,E) Representative images showing tdTomato^+^ expression in PVT of males (D) and females (E); scale bar: 100 µm. Analyzed by unpaired t test; *P < 0.05, **P < 0.01. Data are presented as means ± SEM.

Previous studies have highlighted the crucial role of thalamus in cognition.^[^ ^12^, ^13^, ^14^ ^]^ To determine which thalamic subregion contributes to the memory impairment caused by repeated neonatal anesthesia, we assessed neuronal activity in the thalamus during memory retrieval via a reporter line generated by crossing *TRAP2* mice with *Ai9*‐tdTomato reporter mice (**Figure** **2**A). To screen for potentially relevant thalamic subregions, we compared tdTomato^+^ cells between the control and anesthesia groups to identify differentially regulated brain regions in both sexes.

Interestingly, although mice of both sexes exhibited similar deficits in fear memory, we found sex‐based differences in thalamic neuronal activity. In males, anesthesia‐treated mice exhibited significant decreases in tdTomato^+^ cells in the reuniens nucleus and paraventricular nucleus, and significant increases in the intermediodorsal nucleus and paratenial nucleus (Figure 2B; Figure S3, Supporting Information). In females, significant decreases were observed in the anteromedial nucleus, anteroventral nucleus, paratenial nucleus, and paraventricular nucleus (Figure 2C; Figure S4, Supporting Information). Notably, the PVT was one of the most highly inhibited thalamic regions in both male and female anesthesia‐treated mice (Figure 2D,E), so we focused on it in the further investigations. Neuronal nuclei (NeuN) staining showed no significant neuronal loss in the PVT between the control and anesthesia groups, indicating that the reduced tdTomato^+^ cells was due to decreased neuronal activity rather than cell death (Figure S5, Supporting Information).

**Figure 3:**
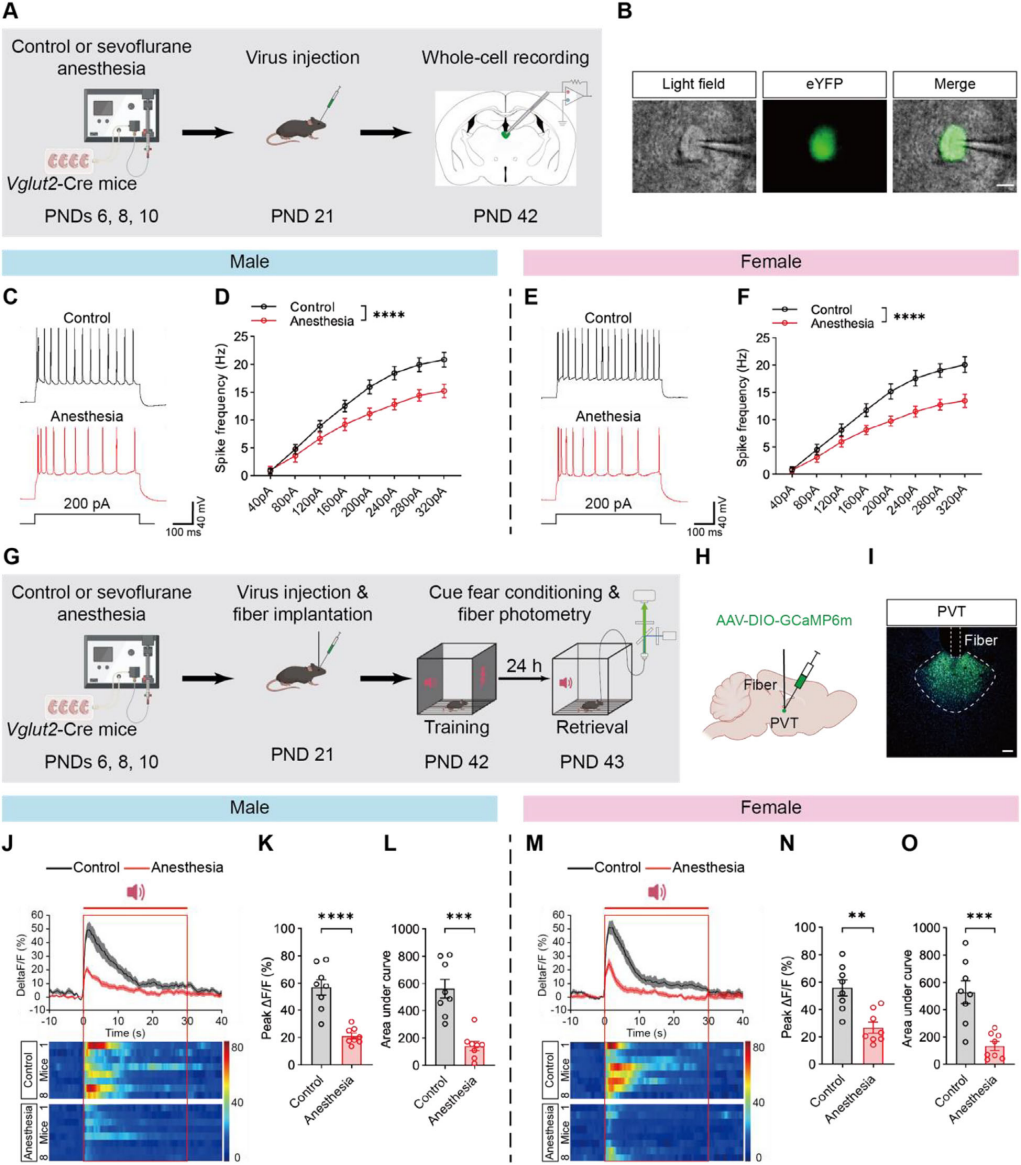
Ex vivo and in vivo recordings of PVT glutamatergic neurons activity. A) Schematic of labeling PVT glutamatergic neurons with virus and ex vivo whole‐cell recordings in PVT slices. B) Representative images of real‐time recordings of PVT glutamatergic neurons; scale bar: 10 µm. C‐F) Representative traces of AP elicited by 200 pA current injections (C, E) and AP frequency in response to a range of current injections (D, F) in males (C, D) and females (E, F); n = 10‐12 neurons from 4 mice per group. G) Schematic of experimental design and timeline of fiber photometry. H,I) Diagram of virus injection and fiber implantation into the PVT (H) and representative image showing virus expression and fiber site (I); scale bar: 100 µm. J,M) Average calcium‐dependent GCaMP fluorescent signal (top) of PVT glutamatergic neurons and the corresponding heatmaps (down) in males (J) and females (M); duration of the tone is indicated by the red box. K,N) Anesthesia group showed significantly reduced peak ΔF/F of calcium activity in the PVT glutamatergic neurons compared to control group in males (K) and females (N); n = 8 mice per group. L,O) Anesthesia group showed significantly decreased the AUC of calcium activity in the PVT glutamatergic neurons compared to control group in males (L) and females (O); n = 8 mice per group. Analyzed by two‐way repeated‐measure ANOVA in D and F, and unpaired t test in J, K, M and N; **P < 0.01, ***P < 0.001, **** *P* < 0.0001. Data are presented as means ± SEM.

Since neurons in the PVT are predominantly glutamatergic without γ‐aminobutyric acid (GABA)ergic interneurons^[^ ^35^, ^36^ ^]^ (Figure S6, Supporting Information), we utilized *Vglut2*‐Cre mice to investigate the role of PVT glutamatergic neurons in anesthesia‐induced memory deficits. To confirm that PVT glutamatergic neurons were inhibited in anesthesia‐treated mice, we performed ex vivo whole‐cell recordings on neurons labeled by injecting AAV‐DIO‐eYFP into the PVT of *Vglut2*‐Cre mice (**Figure** **3**A,B). Whole‐cell recordings revealed that eYFP^+^ neurons from anesthesia‐treated mice exhibited significantly decreased excitability, as evidenced by reduced action potential (AP) firing frequency in males (Figure 3C,D) and females (Figure 3E,F). To further investigate the in vivo dynamics of PVT glutamatergic neurons during fear memory retrieval, we injected AAV‐DIO‐GCaMP6s into the PVT of *Vglut2*‐Cre mice to label PVT glutamatergic neurons (Figure 3G–I). Using fiber photometry, we measured the changes in fluorescent Ca^2+^ activity and found that anesthesia‐treated mice exhibited significantly a lower activity of PVT glutamatergic neurons compared to control mice during memory retrieval in males (Figure 3J–L) and females (Figure 3M–O).

These results suggest that the inhibition of PVT glutamatergic neurons is associated with impaired memory induced by repeated neonatal anesthesia.

### 2.3 PVT Glutamatergic Neurons Modulated the Detrimental Effects of Repeated Neonatal Anesthesia on Fear Memory

**Figure 4:**
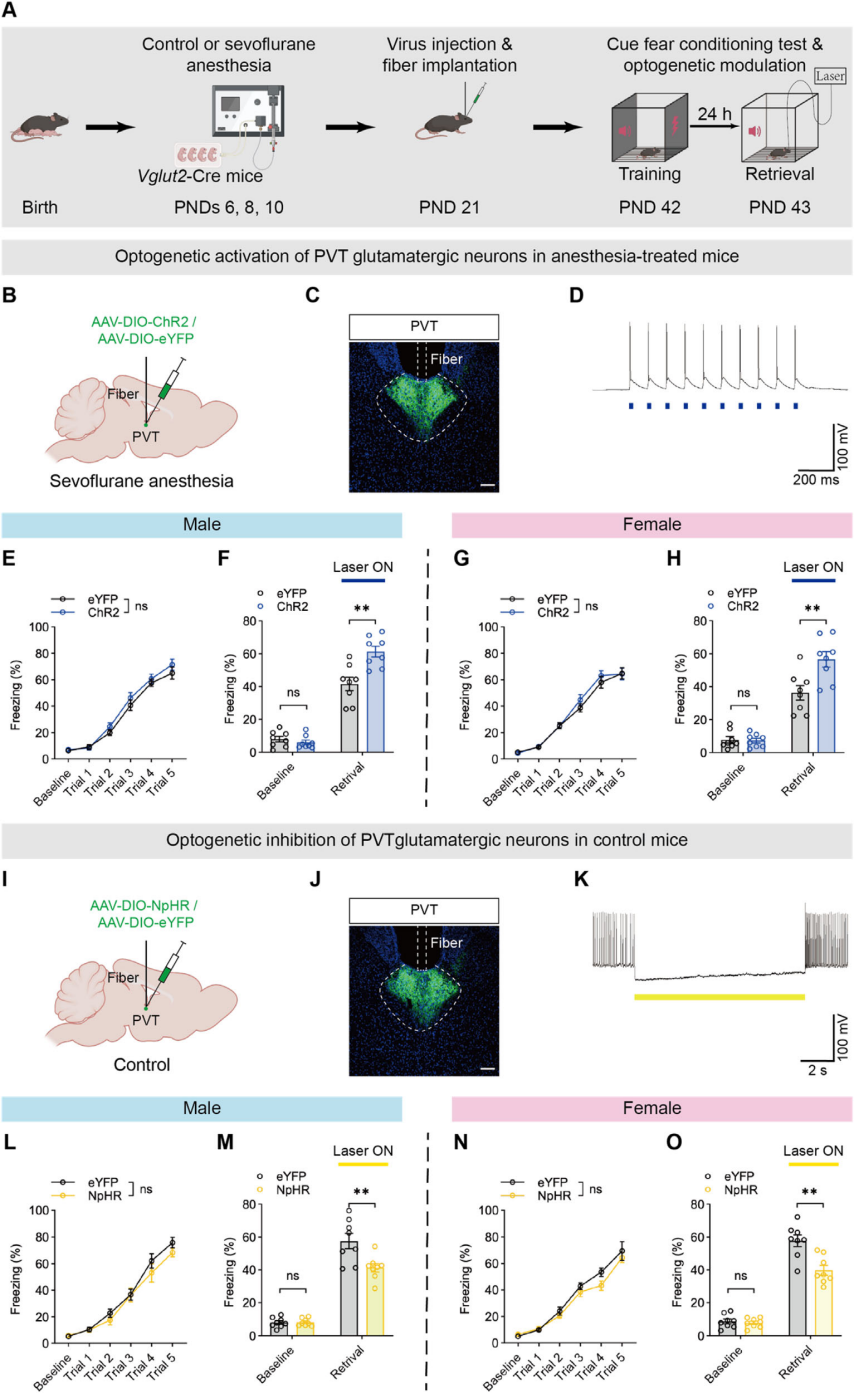
PVT glutamatergic neuron activity drove fear memory impairment induced by repeated neonatal anesthesia. A) Schematic of experimental design and timeline of optogenetic modulation. B,C) Diagram of virus injection and fiber implantation into the PVT of anesthesia‐treated mice (B) and representative image showing virus expression and fiber site (C); scale bar: 100 µm. D) Representative trace of neuronal firing in ChR2‐expressing neurons evoked by 473‐nm photostimulation at 10 Hz. E,G) No significant difference in training performance between eYFP and ChR2 groups in both males (E) and females (G). F,H) Optogenetic activation of PVT glutamatergic neurons ameliorated fear memory impairment in both males (F) and females (H), while baseline freezing levels showed no significant differences in either sex. I,J) Diagram of virus injection and fiber implantation into the PVT of control mice (I) and representative image showing virus expression and fiber site (J); scale bar: 100 µm. K) Representative trace of neuronal firing in NpHR‐expressing neurons suppressed by 590‐nm laser. L,N) No significant difference in training performance between eYFP and NpHR groups in both males (L) and females (N). M,O) Optogenetic inhibition of PVT glutamatergic neurons induced fear memory impairment in both males (M) and females (O), while baseline freezing levels showed no significant differences in either sex. Analyzed by two‐way repeated‐measure ANOVA in E, G, L and N and unpaired t test in F, H, M and O; n = 8 mice per group; **P < 0.01, ns: not significance. Data are presented as means ± SEM.

To determine the role of PVT glutamatergic neurons in the memory impairment induced by repeated neonatal anesthesia, we used optogenetics to manipulate PVT glutamatergic neurons (**Figure** **4**A). We injected either AAV‐EF1a‐DIO‐eYFP or AAV‐EF1a‐DIO‐ChR2‐eYFP into the PVT of *Vglut2*‐Cre anesthesia‐treated mice (Figure 4B). Immunofluorescence confirmed successful transfection of the optogenetic viruses (Figure 4C). Whole‐cell recordings indicated that ChR2‐expressing neurons responded to optical stimulation with a 473‐nm laser (10 Hz, 15‐ms pulse width, and 3.5 mW) (Figure 4D). Both the eYFP and the ChR2 groups demonstrated successful acquisition of fear memories during the training phase, characterized by a gradual increase in the percentage of freezing time in males (Figure 4E) and females (Figure 4G). However, during memory retrieval, the ChR2 group showed a significantly increased percentage of freezing time, suggesting that the activation of PVT glutamatergic neurons in anesthesia‐treated mice ameliorated memory impairment (Figure 4F,H).

To further validate the role of PVT glutamatergic neurons in anesthesia‐induced memory impairment, we injected either AAV‐EF1a‐DIO‐eYFP or AAV‐EF1a‐DIO‐NpHR‐eYFP into the PVT of *Vglut2*‐Cre control mice (Figure 4I,J). Whole‐cell recordings revealed that NpHR‐expressing neurons were suppressed by the optical stimulation with a 594‐nm laser (continuous inhibition, 3.5 mW) (Figure 4K). There was no significant difference in the learning curve between the eYFP and NpHR groups during the training phase in either males (Figure 4L) or females (Figure 4N). During memory retrieval, however, inhibiting PVT glutamatergic neurons in control mice disrupted fear memory (Figure 4M,O), mimicking the detrimental effects of repeated neonatal anesthesia.

In summary, manipulation of PVT glutamatergic neurons modulated the fear memory deficits induced by repeated neonatal anesthesia.

### 2.4 Repeated Neonatal Anesthesia Reduced Igfbp2 Expression in PVT Glutamatergic Neurons

**Figure 5:**
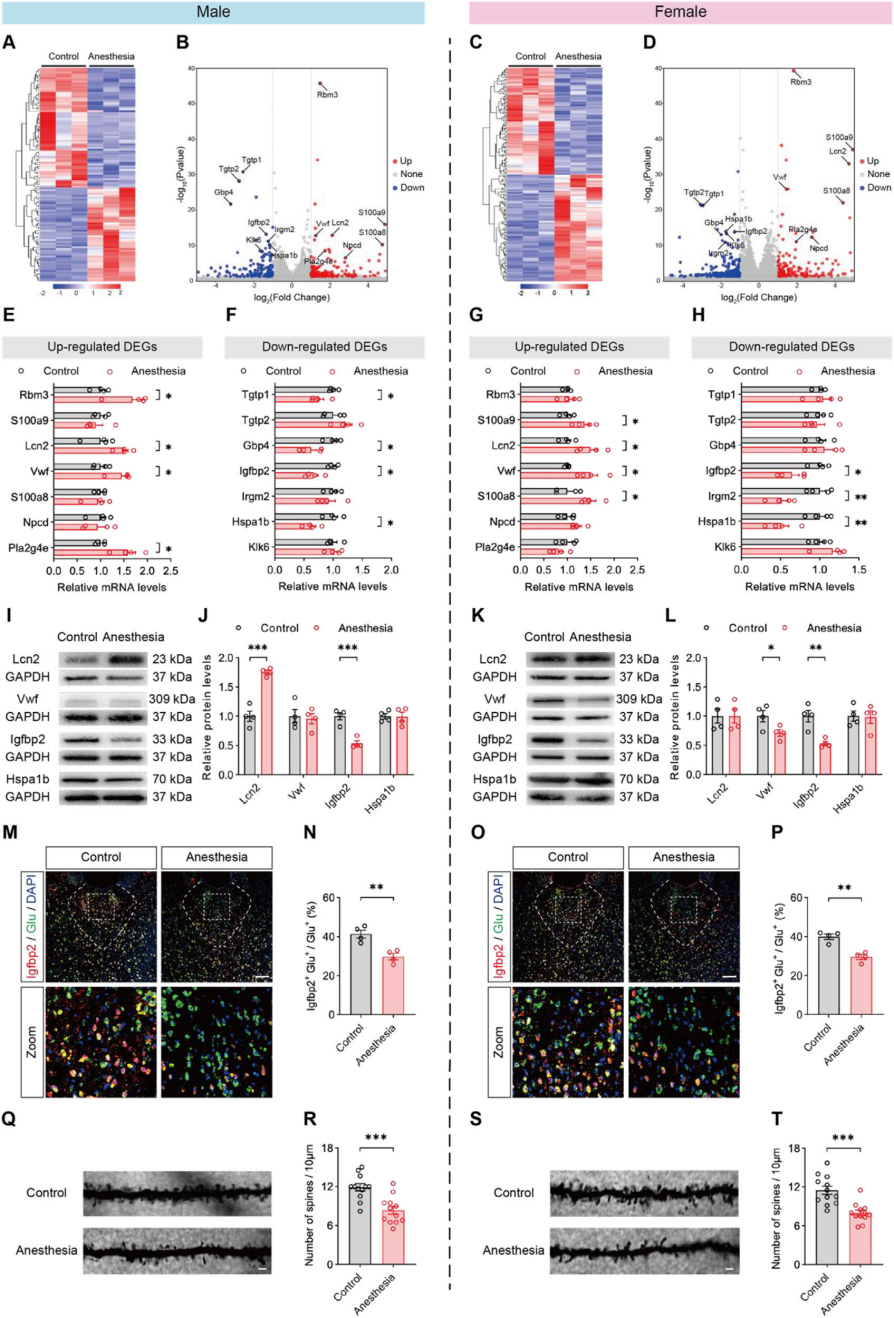
Repeated neonatal anesthesia reduced Igfbp2 expression in PVT glutamatergic neurons and decreased spine density in PVT. A,C) Heatmaps showing DEGs between control and anesthesia groups identified by RNA‐seq of PVT tissues from males (A) and females (C). Normalized Z score values (high, red; low, blue) were calculated for each DEG (row). B,D) Volcano plots illustrating the distribution of upregulated (red), downregulated (blue), and unchanged (gray) DEGs in PVT tissues in males (B) and females (D); each dot represents one gene. E,G) RT‐qPCR of up‐regulated DEGs in males (E) and females (G); n = 4 mice per group. F,H) RT‐qPCR of down‐regulated DEGs in males (F) and females (H); n = 4 mice per group. I‐L) Representative western blot bands (I, K) and quantification analysis of Lcn2, Vwf, Igfbp2 and Hspa1b expression in PVT tissues (J, L) between control and anesthesia groups in males (I, J) and females (K, L); n = 4 mice per group. M‐P) Representative images (M, O) and quantification analysis of the percentage of Igfbp2‐expressing glutamatergic neurons in PVT (N, P) between control and anesthesia groups in males (M, N) and females (O, P); scale bar: 100 µm; n = 4 brain sections from 4 mice per group. Q‐T) Representative Golgi‐Cox staining images (Q, S) and quantification analysis of dendritic spine numbers per 10 µm in PVT (R, T) between control and anesthesia groups in males (Q, R) and females (S, T); scale bar: 5 µm; n=12 brain sections from 4 mice per group. Analyzed by unpaired t test; *P < 0.05, **P < 0.01, ***P < 0.01, ns: not significance. Data are presented as means ± SEM.

To identify key molecules contributing to memory impairment and reduced activity in the PVT of anesthesia‐treated mice, bulk RNA sequencing (RNA‐seq) of PVT tissue was performed. In male mice, a total of 116 differentially expressed genes (DEGs) were identified, comprising 51 up‐ and 65 down‐regulated genes (**Figure** **5**A,B; Table S1, Supporting Information). In female mice, 184 DEGs were identified, including 92 up‐ and 92 down‐regulated genes (Figure 5C,D; Table S2).

Focusing on the top 20 up‐ and down‐regulated DEGs in both sexes, we identified 14 overlapping genes between males and females (Figure S7 and Table S3, Supporting Information). These genes might be critically involved in anesthesia‐induced fear memory deficits. We validated the expression of these 14 genes via real‐time quantitative PCR (RT‐qPCR). Between the males and females, we found two common up‐regulated genes (Lcn2 and Vwf) (Figure 5E,G) and two common down‐regulated genes (Igfbp2 and Hspa1b) (Figure 5F,H). Western blot confirmed that among these genes, the protein expression of Igfbp2 was significantly lower in the anesthesia group in both sexes (Figure 5I–L). Although Igfbp2 has been reported to be implicated in learning and memory in the prefrontal cortex, hippocampus, and amygdala,^[^ ^30^, ^31^ ^]^ we did not detect significant changes in Igfbp2 protein levels in these regions following repeated neonatal anesthesia (Figure S8, Supporting Information).

Immunofluorescence revealed that in the PVT, Igfbp2 was predominantly coexpressed with Glu (glutamatergic neuron marker) and sparsely coexpressed with either GFAP (astrocyte marker) or IBA1 (microglia marker) (Figure S9, Supporting Information). Importantly, the Igfbp2 expression levels in the PVT glutamatergic neurons were significantly reduced in the anesthesia‐treated mice in both sexes (Figure 5M–P). Previous studies have shown that Igfbp2 affected dendritic spine density.^[^ ^29^, ^37^, ^38^ ^]^ We performed Golgi staining and found that spine density in the PVT was reduced in the anesthesia group compared to the control group in both sexes (Figure 5Q–T).

These results suggested that Igfbp2 in PVT glutamatergic neurons played a pivotal role in the detrimental effects induced by repeated neonatal anesthesia.

### 2.5 PVT Glutamatergic Igfbp2 Contributed to Fear Memory Deficits Caused by Repeated Neonatal Anesthesia by Modulating Neuron Excitability and Synaptic Density

**Figure 6:**
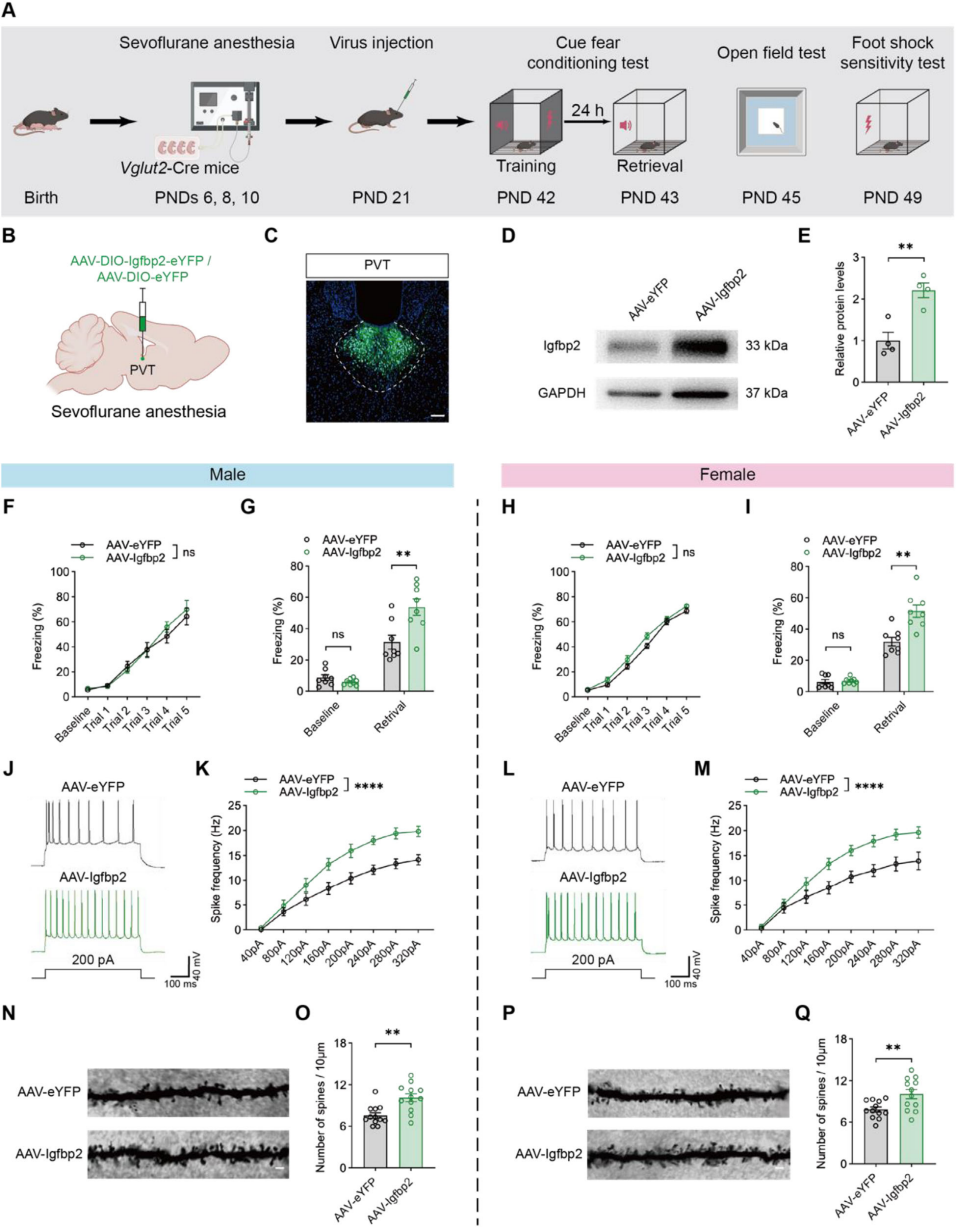
Overexpression of Igfbp2 in PVT glutamatergic neurons rescued fear memory deficits, neuronal excitability impairments, and spine loss induced by repeated neonatal anesthesia. A) Schematic of experimental design and timeline of Igfbp2 overexpression in PVT glutamatergic neurons. B,C) Diagram of virus injection into PVT of anesthesia‐treated mice (B) and representative image showing virus expression (C); scale bar: 100 µm. D,E) Representative western blot bands (D) and quantification analysis of Igfbp2 in PVT (E) between AAV‐eYFP and AAV‐Igfbp2 groups; n = 4 mice per group. F,H) No significant difference in training performance between AAV‐eYFP and AAV‐Igfbp2 groups in males (F) and females (H); n = 8 mice per group. G,I) Restoration of Igfbp2 in PVT glutamatergic neurons rescued fear memory impairment in males (G) and females (I), while baseline freezing levels showed no significant differences in either sex; n = 8 mice per group. J‐M) Representative traces of AP elicited by 200 pA current injections (J, L), and AP frequency in response to a range of current injections (K, M) between AAV‐eYFP and AAV‐Igfbp2 groups in males (J, K) and females (L, M); n = 10 neurons from 4 mice per group. N‐Q) Representative Golgi‐Cox staining images (N, P) and quantification analysis of dendritic spine numbers per 10 µm in PVT (O, Q) between AAV‐eYFP and AAV‐Igfbp2 groups in males (N, O) and females (P, Q); scale bar: 5 µm; n = 12 brain sections from 4 mice per group. Analyzed by two‐way repeated‐measure ANOVA in F and H, and unpaired t test in G, I, K, M, O and Q; *P < 0.05, **P < 0.01, ns: not significance. Data are presented as means ± SEM.

To determine whether Igfbp2 in PVT glutamatergic neurons plays a pivotal role in anesthesia‐induced memory deficits, Igfbp2 was specifically overexpressed in these neurons by injecting AAV‐EF1a‐DIO‐Igfbp2‐eYFP (AAV‐Igfbp2) into the PVT of *Vglut2*‐Cre anesthesia‐treated mice (**Figure** **6**A,B). AAV‐EF1a‐DIO‐eYFP (AAV‐eYFP) was used as a negative control. The efficiency of the overexpression was confirmed by immunostaining and western blot analysis (Figure 6C–E). No significant difference in the learning curves was observed between the AAV‐eYFP and the AAV‐Igfbp2 groups in either males (Figure 6F) or females (Figure 6H). Notably, Igfbp2 overexpression rescued the fear memory behaviors of the anesthetized mice of both sexes (Figure 6G,I). Igfbp2 overexpression also restored neuronal excitability in the PVT glutamatergic neurons (Figure 6J–M) and increased spine density in the PVT (Figure 6N–Q). Importantly, Igfbp2 overexpression did not affect locomotor activity or foot‐shock thresholds, nor did it induce anxiety‐like behavior (Figure S10, Supporting Information).

**Figure 7:**
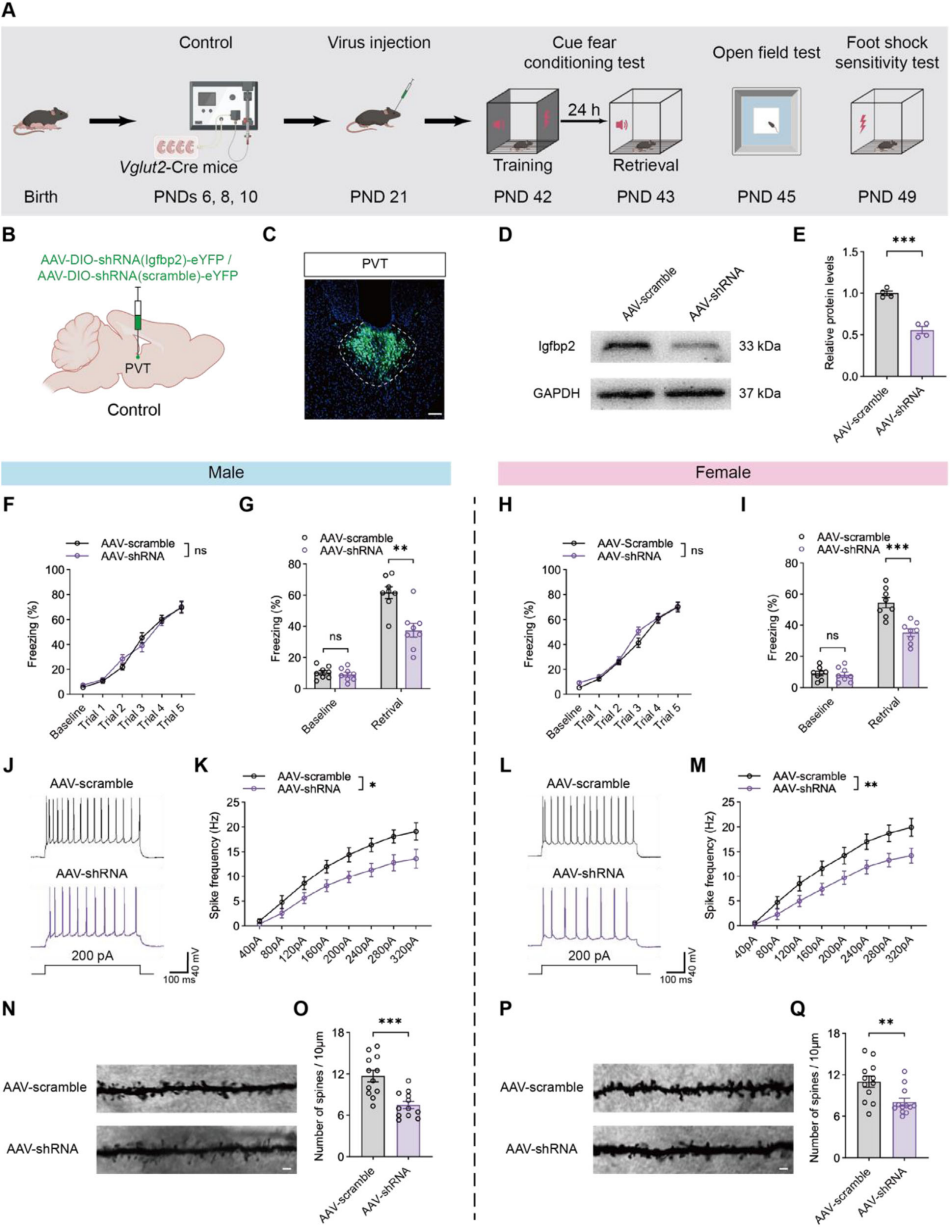
Knockdown of Igfbp2 in PVT glutamatergic neurons impaired fear memory retrieval, neuronal excitability and spine density. A) Schematic of experimental design and timeline of Igfbp2 knockdown in PVT glutamatergic neurons. B,C) Diagram of virus injection into PVT of control mice (B) and representative image showing virus expression (C); scale bar: 100 µm. D,E) Representative western blot bands (D) and quantification analysis of Igfbp2 in PVT (E) between AAV‐scramble and AAV‐shRNA groups; n = 4 mice per group. F,H) No significant difference in training performance between AAV‐scramble and AAV‐shRNA groups in males (F) and females (H); n = 8 mice per group. G,I) Knockdown of Igfbp2 in PVT glutamatergic neurons induced fear memory impairment in males (G) and females (I), while baseline freezing levels showed no significant differences in either sex; n = 8 mice per group. J‐M) Representative traces of AP elicited by 200 pA current injections (J, L), and AP frequency in response to a range of current injections (K, M) between AAV‐scramble and AAV‐shRNA groups in males (J, K) and females (L, M); n =10 neurons from 4 mice per group. N‐Q) Representative Golgi‐Cox staining images (N, P) and quantification of dendritic spine numbers per 10 mm in PVT (O, Q) from AAV‐scramble and AAV‐shRNA groups in males (N, O) and females (P, Q); scale bar: 5 µm; n = 12 brain sections from 4 mice per group. Analyzed by two‐way repeated‐measure ANOVA in F and H, and unpaired t test in G, I, K, M, O and Q; *P < 0.05, **P < 0.01, ns: not significance. Data are presented as means ± SEM.

To further explore the impact of Igfbp2 on anesthesia‐induced memory impairment, we injected AAV‐CMV‐DIO‐shRNA (Igfbp2)‐eYFP (AAV‐shRNA) or the scramble virus AAV‐CMV‐DIO‐shRNA (scramble)‐eYFP (AAV‐scramble) into the PVT of *Vglut2*‐Cre control mice (**Figure** **7**A,B). The knockdown efficiency was confirmed by immunostaining and western blot (Figure 7C–E). Despite of no significant differences in learning curves between the groups (Figure 7F,H), the AAV‐shRNA group showed significant fear memory impairment compared to the AAV‐scramble group in both sexes (Figure 7G,I). Downregulation of Igfbp2 in the PVT glutamatergic neurons decreased the neuronal excitability (Figure 7J‐M) and reduced spine density in the PVT (Figure 7N–Q). Notably, Igfbp2 downregulation did not affect locomotor activity, foot shock thresholds, or induce anxiety‐like behavior (Figure S11, Supporting Information), nor did it alter the number of the PVT neurons (Figure S12, Supporting Information).

These findings identified Igfbp2 in the PVT glutamatergic neurons as a key mediator of anesthesia‐induced memory impairment, primarily through its impact on neuronal excitability and synaptic density.

### 2.6 Igfbp2 in the PVT Glutamatergic Neurons Modulated Fear Memory Deficits Induced by Repeated Neonatal Anesthesia via Projections to the CeA

**Figure 8:**
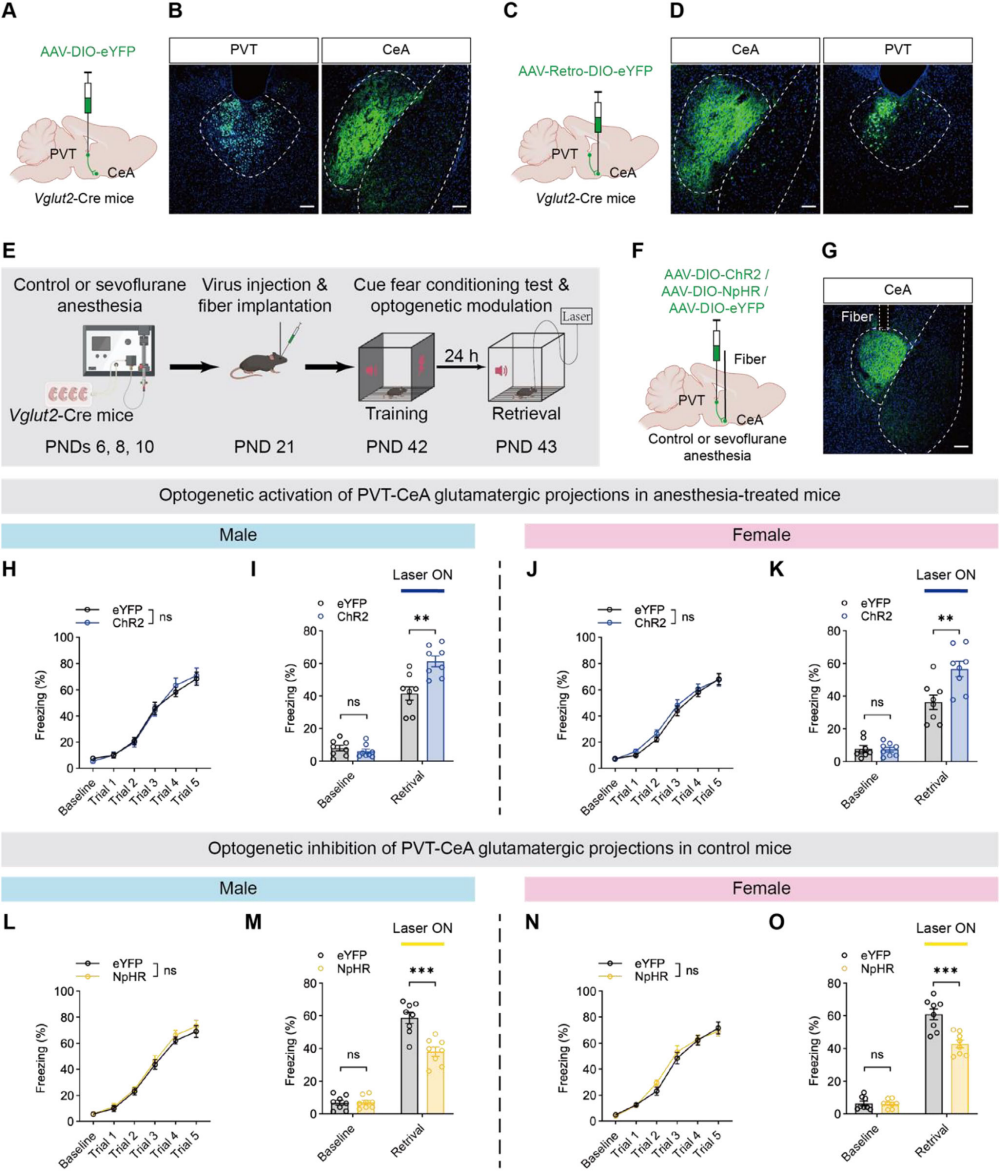
PVT‐CeA glutamatergic projections contributed to fear memory impairment induced by repeated neonatal anesthesia. A,B) Schematic of anterograde virus tracing (A) and representative images showing virus injection site within PVT (left) and virus expression in CeA (right) (B); scale bars: 100 µm. C,D) Schematic of retrograde virus tracing (C) and representative images of virus injection site within CeA (left) and virus expression in PVT (right) (D); scale bars: 100 µm. E) Schematic of experimental design and timeline of optogenetic modulation. F,G) Diagram of virus injection and fiber implantation (F) and representative image showing virus expression and fiber site in CeA (G); scale bars: 100 µm. H,J) No significant difference in training performance between eYFP and ChR2 groups in anesthesia‐treated males (H) and females (J). I,K) Optogenetic activation of PVT‐CeA glutamatergic projections ameliorated fear memory impairment in anesthesia‐treated males (I) and females (K), while baseline freezing levels showed no significant differences in either sex. L,N) No significant difference in training performance between eYFP and NpHR groups in control males (L) and females (N). M,O) Optogenetic inhibition of PVT‐CeA glutamatergic projections induced fear memory deficits in control males (M) and females (O), while baseline freezing levels showed no significant differences in either sex. Analyzed by two‐way repeated‐measure ANOVA in H, I, L and N, and unpaired t test in I, K, M and O; n = 8 mice per group; **P < 0.01, ***P < 0.001, ns: not significance. Data are presented as means ± SEM.

To explore the specific downstream projections of the PVT glutamatergic neurons in anesthesia‐induced memory impairment, the anterograde tracer AAV‐DIO‐eYFP was injected into the PVT of *Vglut2*‐Cre mice to specifically label glutamatergic neurons (**Figure** **8**A). Notably, eYFP^+^ axonal fibers were detected mainly in the CeA (Figure 8B; Figure S13, Supporting Information). As previous studies have highlighted the critical role of CeA in fear memory,^[^ ^39^, ^40^ ^]^ we investigated whether the PVT‐CeA projections were involved in our model. To confirm this projection, we injected the retrograde tracer virus AAV‐Retro‐DIO‐eYFP into the CeA of *Vglut2*‐Cre mice and observed strong eYFP fluorescence in the PVT (Figure 8C,D).

Next, we used optogenetics to selectively manipulate glutamatergic projections from the PVT to the CeA (Figure 8E–G). Specifically, we injected AAV‐EF1a‐DIO‐ChR2‐eYFP or AAV‐EF1a‐DIO‐eYFP into the PVT of *Vglut2*‐Cre anesthesia‐treated mice, and optical fibers were bilaterally implanted above the CeA. No significant difference was observed in the learning curve between the eYFP and ChR2 groups during the training phase in either sex (Figure 8H,J). However, optogenetic activation of these projections in ChR2‐expressing mice significantly increased the percentage of freezing time during memory retrieval in both sexes (Figure 8I,K). Furthermore, we expressed AAV‐EF1a‐DIO‐NpHR‐eYFP or AAV‐EF1a‐DIO‐eYFP in the PVT of *Vglut2*‐Cre control mice. Both the eYFP and the NpHR groups successfully acquired fear memories during the training phase in both sexes (Figure 8L,N). During the memory retrieval phase, optogenetic inhibition of the projections in NpHR‐expressing mice reduced freezing behavior (Figure 8M,O).

**Figure 9:**
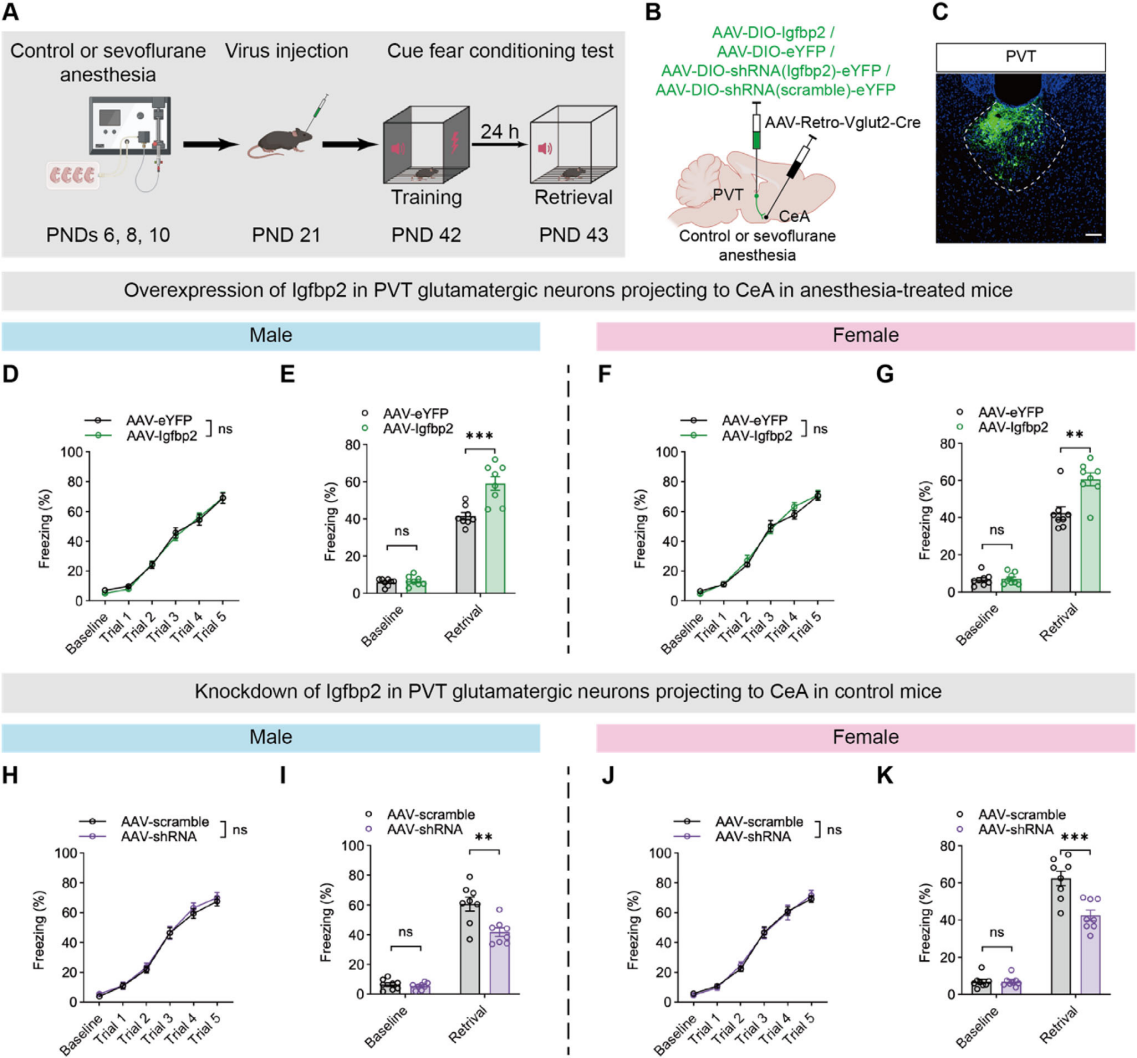
Igfbp2 in glutamatergic neurons of PVT projecting to CeA modulated fear memory impairment induced by repeated neonatal anesthesia. A) Schematic of experimental design and timeline of specific modulation of Igfbp2 in PVT glutamatergic neurons projecting to CeA. B,C) Diagram of virus injection into PVT (B) and representative image showing virus expression (C); scale bars: 100 µm. D,F) No significant difference in training performance between AAV‐eYFP and AAV‐Igfbp2 groups in anesthesia‐treated males (D) and females (F). E,G) Restoration of Igfbp2 expression in PVT glutamatergic neurons projecting to CeA rescued fear memory impairment in anesthesia‐treated males (E) and females (G), while baseline freezing levels showed no significant difference in either sex. H,J) No significant difference in training performance between AAV‐scramble and AAV‐shRNA groups in control males (H) and females (J). I,K) Knockdown of Igfbp2 in PVT glutamatergic neurons projecting to CeA induced fear memory deficits in control males (I) and females (K), while baseline freezing levels showed no significant differences in either sex. Analyzed by two‐way repeated‐measure ANOVA in D, F, H and J, and unpaired t test in E, G, I and K; n = 8 mice per group; **P < 0.01, ***P < 0.001, ns: not significance. Data are presented as means ± SEM.

We next investigated whether Igfbp2 expression in PVT glutamatergic neurons projecting to the CeA contributes to the fear memory impairment induced by repeated neonatal anesthesia. Using immunofluorescence staining combined with retrograde tracing, we found that ≈24.15% of CeA‐projecting PVT neurons co‐expressed Igfbp2 (Figure S14, Supporting Information). Based on this anatomical evidence, we then selectively modulated Igfbp2 expression in these projection‐specific PVT glutamatergic neurons to determine its causal role in anesthesia‐induced memory impairment (**Figure** **9**A–C). We injected AAV‐EF1a‐DIO‐Igfbp2‐eYFP (AAV‐Igfbp2) or AAV‐EF1a‐DIO‐eYFP (AAV‐eYFP) into the PVT and AAV‐Retro‐Vglut2‐Cre bilaterally into the CeA of wild‐type anesthesia‐treated mice. Igfbp2 overexpression in the PVT‐CeA projections led to an increased percentage of freezing time during memory retrieval in the AAV‐Igfbp2 group compared to the AAV‐eYFP group in both sexes (Figure 9E,G), whereas no significant difference in the learning curves between the groups (Figure 9D,F). In addition, we knocked down the expression of Igfbp2 in the PVT‐CeA projections by injecting AAV‐CMV‐DIO‐shRNA (Igfbp2)‐eYFP (AAV‐shRNA) or AAV‐CMV‐DIO‐shRNA (scramble)‐eYFP (AAV‐scramble) into the PVT and AAV‐Retro‐Vglut2‐Cre into the CeA of wild‐type control mice. The learning curves did not differ significantly between the AAV‐shRNA and AAV‐scramble groups in either sex (Figure 9H,J). However, the AAV‐shRNA group showed notable fear memory impairment compared to the AAV‐scramble group in both sexes (Figure 9I,K).

These findings suggest that Igfbp2 modulated memory impairment induced by repeated neonatal anesthesia through the PVT glutamatergic neurons projecting to the CeA.

## 3 Discussion

**Figure 10:**
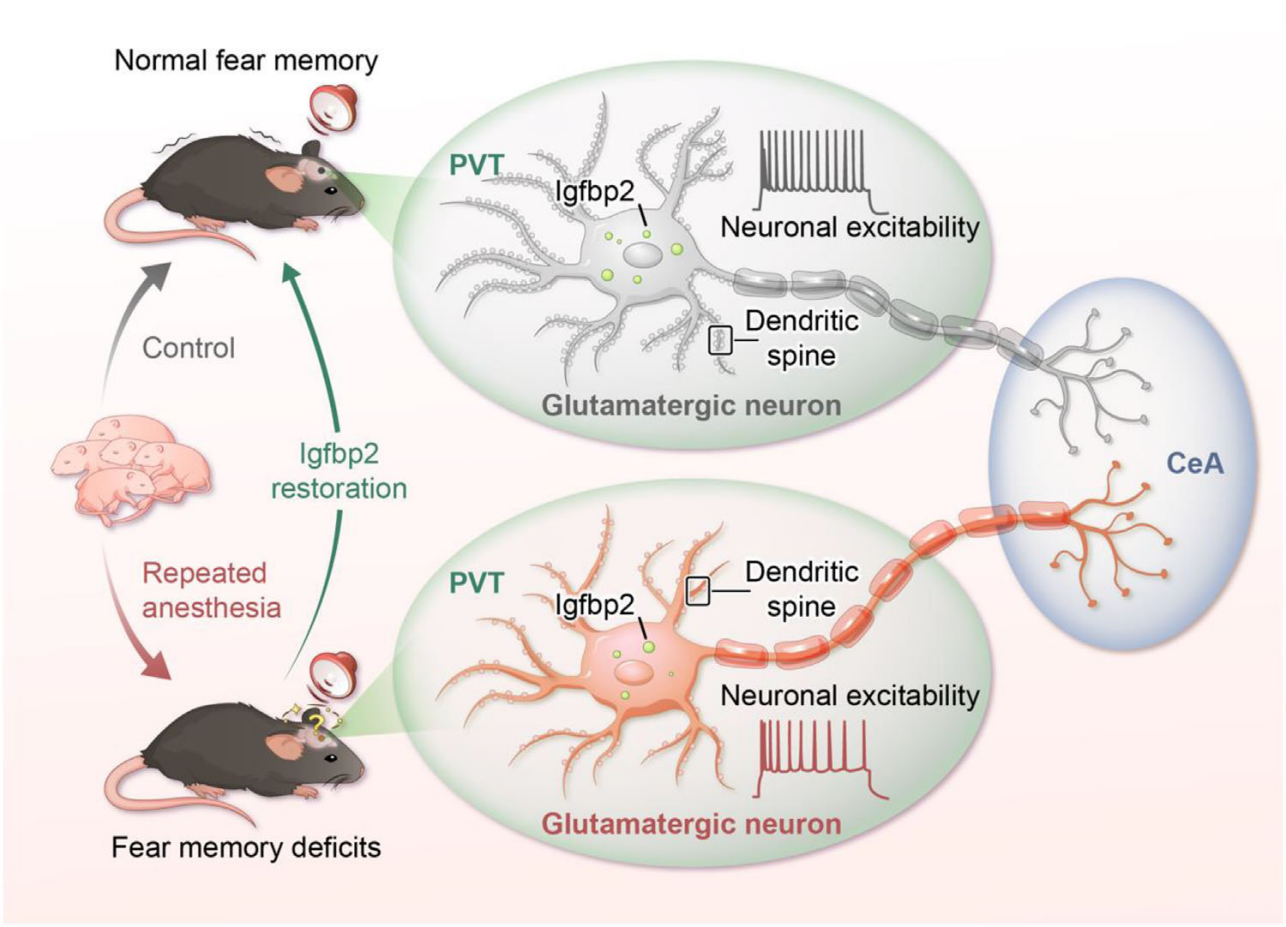
Schematic summary of this study. Repeated neonatal anesthesia impaired fear memory in adolescent mice of both sexes, accompanied by reduced PVT glutamatergic neuron activity and downregulated Igfbp2 expression. Manipulating Igfbp2 expression in PVT glutamatergic neurons mediated anesthesia‐induced fear memory deficits by modulating neuronal excitability and dendritic spine density. Selectively optogenetic activation or restoration of Igfbp2 expression in PVT‐CeA glutamatergic projections ameliorated anesthesia‐induced memory deficits, while optogenetic inhibition or Igfbp2 knockdown in these projections recapitulated memory deficits in control mice. PVT paraventricular thalamus, CeA central amygdala, Igfbp2 Insulin‐like growth factor‐binding protein 2.

In this study, we found that the reduced expression of Igfbp2 in the PVT glutamatergic neurons contributed to fear memory deficits caused by repeated neonatal anesthesia in adolescent mice by modulating neuronal activity and spine density. Additionally, our findings demonstrated that Igfbp2 expression in the PVT glutamatergic neurons projecting to the CeA was crucial for mediating these anesthesia‐induced memory deficits (**Figure** **10**).

We administered general anesthesia with sevoflurane, a commonly used general anesthetic for pediatric patients, to neonatal male and female mice for 2 h per day on PNDs 6, 8, and 10. The selection of this anesthesia protocol was based on the previous studies in which animal models were established to mimic the effects of repeated general anesthesia exposure in children.^[^ ^41^, ^42^, ^43^ ^]^ Considering the weaning age and lifespan of mice, the period of anesthetic intervention in our study corresponds to the developmental stage of ≈6 to 12 months of age in human infants.^[^ ^44^ ^]^ This stage represents a peak period of brain development, during which the central nervous system is particularly vulnerable to external insults. Therefore, it provides a critical window for investigating the potential impact of repeated anesthesia exposure on early childhood brain development.

Previous animal research has predominantly focused on changes in the hippocampus during anesthesia‐induced neurodevelopmental toxicity, but the potential involvement of the thalamus remains poorly understood. We observed consistent hypoactivation of the PVT in anesthesia‐treated mice of both sexes by mapping thalamic neuronal activity during fear memory retrieval in *TRAP2/Ai9* mice. These findings suggest that the PVT may play a pivotal role in anesthesia‐induced memory impairment. Interestingly, sex‐specific neuronal activity was also observed in other thalamic regions, despite both sexes exhibiting similar impairments in fear memory following repeated neonatal anesthesia. Previous studies have highlighted sex‐dependent differences in the mechanisms underlying fear memory processing, including differential engagement of neural circuits, as well as variations in synaptic plasticity, gene expression, and stress hormone responsiveness between males and females.^[^ ^45^, ^46^, ^47^ ^]^ Moreover, sex hormones such as estrogen and testosterone play critical roles in the development and function of neural circuits involved in fear memory.^[^ ^48^, ^49^ ^]^ These hormonal effects may shape how male and female brains respond to repeated neonatal anesthesia, potentially resulting in distinct neurobiological adaptations even when behavioral outcomes appear similar. Collectively, these factors may account for the dissociation observed in our study between sex‐specific patterns of thalamic neuronal activity and convergent behavioral outcomes. Future studies are needed to clarify the specific contributions of these distinct thalamic subregions to anesthesia‐induced memory impairments. In particular, employing more refined and multidimensional behavioral assessments may help uncover subtle sex‐specific differences that were not detected in the present study.

Through ex vivo whole‐cell recordings, we found that repeated anesthesia decreased the excitability of glutamatergic neurons in the PVT of anesthesia‐treated mice. This finding was further supported by in vivo fiber photometry recordings. Optogenetic activation of the PVT glutamatergic neurons effectively alleviated the adverse effects of anesthesia‐induced fear memory impairment, whereas inhibition of these neurons during memory retrieval in control mice disrupted fear memory, which is consistent with previous studies.^[^ ^27^, ^50^ ^]^ These results suggest that the PVT is particularly vulnerable to repeated neonatal anesthesia. Importantly, our present study is the first to demonstrate that repeated neonatal anesthesia inhibited glutamatergic neurons in the PVT, contributing to memory impairment in adolescent mice.

The mRNA sequencing of the PVT revealed a significant downregulation of the Igfbp2 gene in both male and female mice after repeated neonatal anesthesia. Additionally, we confirmed that Igfbp2 protein levels in PVT glutamatergic neurons were significantly reduced in anesthesia‐treated mice of both sexes. A recent study underscored the crucial role of hippocampal neuronal Igfbp2 in cognitive development during early life,^[^ ^30^ ^]^ and Igfbp2 was found to significantly reduce neuronal loss and improve functional recovery in a mouse model of spinal cord injury.^[^ ^51^ ^]^ Furthermore, Igfbp2 has been shown to have potential therapeutic benefits for Marfan syndrome and posttraumatic stress disorder by enhancing synaptic plasticity.^[^ ^37^, ^38^ ^]^ Intriguingly, we also observed synaptic plasticity damage in anesthesia‐treated mice, as evidenced by decreased spine density in the PVT. While some studies have highlighted the importance of Igfbp2 for learning and memory in the prefrontal cortex, hippocampus, and amygdala, we did not detect any changes in Igfbp2 protein levels in these regions.^[^ ^30^, ^31^ ^]^ PVT glutamatergic neurons in neonatal rodents are known to be particularly sensitive to early‐life stressors, which may render them more vulnerable to anesthesia‐induced perturbations in Igfbp2 expression.^[^ ^23^ ^]^ In contrast, other brain regions such as the hippocampus and amygdala may rely on distinct regulatory mechanisms or possess compensatory pathways that help maintain stable Igfbp2 levels in the face of anesthetic exposure. This regional specificity may contribute to the selective Igfbp2 downregulation observed in our study. Therefore, the Igfbp2 protein in PVT glutamatergic neurons may be a key molecule underlying anesthesia‐induced developmental neurotoxicity.

Next, we overexpressed Igfbp2 in the PVT glutamatergic neurons in the anesthesia‐treated mice and found that Igfbp2 overexpression not only rescued anesthesia‐induced memory deficits but also improved neuronal excitability and mitigated synaptic damage. To further explore the role of Igfbp2 during fear memory impairment, we knocked down Igfbp2 expression in the PVT glutamatergic neurons in the control mice, showing that Igfbp2 knockdown led to significant deficits in fear memory expression without affecting the acquisition of fear memory. Additionally, the knockdown of Igfbp2 in the PVT glutamatergic neurons decreased the excitability of these neurons and induced dendritic spine loss in the PVT. As a modulator of insulin‐like growth factor (IGF) signaling, Igfbp2 binds to IGFs to regulate their bioavailability and localization.^[^ ^28^ ^]^ A recent study has shown that Igfbp2 can potentiate IGF2 receptor signaling, leading to activation of downstream ERK1/2 pathways that promote dendritic spine maturation and upregulate the expression of GluA1 (an AMPA receptor subunit), along with postsynaptic scaffold proteins such as PSD95 and SAP97 in mouse forebrain neurons.^[^ ^29^ ^]^ Given that dendritic spine density is closely associated with neuronal excitability, Igfbp2 may influence excitability by regulating spine formation and synaptic connectivity. A recent study demonstrated that deletion of Igfbp2 in hippocampal pyramidal neurons during early postnatal development led to reduced input resistance, elevated action potential threshold, and decreased spontaneous firing rates.^[^ ^30^ ^]^ Consistently, another study reported that astrocyte‐derived Igfbp2 enhances the intrinsic excitability of pyramidal neurons by increasing input resistance and lowering the action potential threshold, thereby promoting neuronal firing.^[^ ^52^ ^]^ Additionally, our RNA‐seq data revealed significant alterations in the expression of several ion channel‐related genes (e.g., Kcng4, Kcnj13) and genes involved in glutamate transport and metabolism (e.g., Slc38a2) in the PVT of anesthesia‐treated mice. These findings suggest that Igfbp2 may further modulate neuronal excitability through the regulation of ion channel activity and glutamatergic signaling pathways. Together, these mechanisms may underlie the Igfbp2‐dependent modulation of spine density and neural excitability observed in our study. Interestingly, a recent study revealed that Igfbp2 in BLA astrocytes is crucial for fear memory expression, whereas the knockdown of Igfbp2 led to significant deficits in fear memory without affecting its acquisition.^[^ ^31^ ^]^ Furthermore, the open field test revealed no alterations following the deletion of Igfbp2 in BLA astrocytes, suggesting that Igfbp2 knockdown specifically impacted fear memory processes without influencing general locomotor or anxiety‐related behaviors, which is consistent with our results. Overall, our study demonstrate that Igfbp2 in PVT glutamatergic neurons play a critical role in mediating anesthesia‐induced neurodevelopmental toxicity, primarily through the modulation of neuronal activity and dendritic spine density.

In addition, the viral tracing has shown that PVT glutamatergic neurons send dense afferent projections to the CeA. Previous studies have shown that the PVT‐CeA circuit is crucial for fear memory expression.^[^ ^27^, ^39^ ^]^ Therefore, we explored the role of the PVT‐CeA circuit in anesthesia‐induced memory deficits. We found that bilaterally optogenetic activation of glutamatergic projections in the CeA from the PVT rescued anesthesia‐induced fear memory deficits, while inhibiting these projections impeded fear memory expression. Accumulating evidence supports the involvement of the CeA in the regulation of defensive behaviors, including freezing, flight, and other stress‐related responses.^[^ ^53^, ^54^, ^55^ ^]^ A recent study demonstrated that a brainstem‐CeA circuit mediates defensive responses to learned threats, highlighting the critical role of the CeA in behavioral adaptation and emotional learning.^[^ ^56^ ^]^ Notably, recent studies have also implicated the CeA as a key structure in the processing of fear memory.^[^ ^57^, ^58^ ^]^ Although our study did not directly examine the role of CeA in defense‐related behaviors, our findings clearly indicate that the CeA contributes to the modulation of anesthesia‐induced fear memory impairment. To further investigate whether Igfbp2 in PVT glutamatergic neurons projecting to the CeA underlies anesthesia‐induced memory deficits, we selectively overexpressed Igfbp2 in these neurons. We found that the overexpression of Igfbp2 in PVT glutamatergic neurons projecting to the CeA effectively rescued anesthesia‐induced fear memory deficits. In contrast, selective knockdown of Igfbp2 in the same neurons impaired fear memory expression. These results demonstrate that downregulation of Igfbp2 in glutamatergic neurons in the PVT projecting to the CeA modulates fear memory deficits induced by repeated neonatal anesthesia.

There are several limitations of this study. First, while our study identified Igfbp2 as a key mediator in anesthesia‐induced cognitive impairment, the specific signaling pathways and molecular mechanisms through which Igfbp2 affected neuronal excitability and synaptic function warrant further research. Second, we investigated only the CeA region with projections from PVT glutamatergic neurons in the context of anesthesia‐induced memory impairment; however, further investigations are required to determine the specific types of CeA neurons that receive direct PVT projections. Third, while biologically meaningful transcriptional differences were observed, the relatively small sample size for RNA‐seq validation may limit generalizability. Larger cohorts in future studies will be important for confirming and extending these results. Finally, we primarily focused on fear memory to assess cognitive deficits. While fear conditioning is a robust measure of certain types of memory, it does not encompass the full spectrum of cognitive functions that could be affected by repeated neonatal anesthesia.

In conclusion, our findings demonstrate that Igfbp2 in the PVT glutamatergic neurons projecting to the CeA plays a crucial role in mediating anesthesia‐induced long‐term cognitive impairment in mice of both sexes.

## 4 Experimental Section

### Animals

C57BL/6J mice (8 weeks old, 20–25 g) were purchased from Slaccas Laboratory (China). *Vglut2*‐Cre mice were provided by Dr. Guangyin Xu (Soochow University), whereas *TRAP2* (*FosCreERT2*) mice and *Ai9* mice were obtained from Min Yan (Zhejiang University). The *TRAP2* mice were crossed with *Ai9* mice to generate the double heterozygous mice used in these experiments. Both male and female mice were used in our study. The animals were housed in specific pathogen‐free facilities, with ad libitum food and water access under standard conditions (23–25 °C, 40–60% humidity, 12‐h/12‐h light/dark cycle). For all experiments, mice were randomly assigned to the treatment groups, with sample sizes based on prior behavioral, molecular, and electrophysiological data from our lab.^[^ ^41^ ^]^ The researchers were blinded to genotype and treatment during data acquisition and analysis. All animal procedures followed the Soochow University Ethics Committee's guidelines (Suzhou, Jiangsu, China; approval No.: 202210A0168) and adhered to the Animal Research: Reporting In Vivo Experiments (ARRIVE) guidelines.

### Anesthesia Administration

In this study, male and female offsprings were randomly assigned to the treatment groups. The details of this animal model were previously described.^[^ ^41^ ^]^ Briefly, the anesthesia group received 3% sevoflurane in 60% oxygen, balanced with nitrogen, for 2 h per day on PNDs 6, 8, and 10, while the control group underwent sham exposure (60% oxygen balanced with nitrogen, no sevoflurane) for the same duration. The 3% sevoflurane concentration was the most commonly used concentration for pediatric anesthesia in clinical practice,^[^ ^59^ ^]^ and the sevoflurane concentration was monitored and adjusted with a gas analyzer (Vamos; Dräger Medical, Germany). Each mouse's rectal temperature was maintained at 37 ± 0.5 °C using a heating pad. After treatment, the mice were returned to their home cages. This model has been shown not to affect blood gas or electrolyte levels.^[^ ^42^ ^]^

### Behavioral Tests

The cue fear conditioning test, open field test, and foot shock sensitivity test were conducted from PND 41 to PND 45, as previously described.^[^ ^42^, ^60^ ^]^ The mice were acclimated to the testing environment for 1 h before the behavioral tests, which were performed and analyzed in a blinded manner. All behavioral experiments were conducted during the light phase, specifically between Zeitgeber Time (ZT) 2‐5.

### Cue Fear Conditioning

All cue fear conditioning procedures were performed via Xmaze software (XinRuan Informatics Co. China) according to a previous study.^[^ ^60^ ^]^ Before each session, the cage was cleaned with 70% ethanol. Freezing was defined as no movement for 2 s, and freezing behavior was quantified as the percentage of time spent immobile. The conditioning trial involved a 5‐min exploration period, followed by five paired conditioned stimulus (CS) and unconditioned stimulus (US) trials with a 60‐s interstimulus interval. The CS was a 30‐s, 4‐kHz, 80‐dB tone, and the US was a 1‐s, 0.8‐mA foot shock delivered at the end of the CS. Fear memory was tested 24 h later, with the mice placed in a new context and exposed to two CS presentations, with a 120‐s interstimulus interval after a 5‐min habituation period. Fear memory expression was quantified as the average percentage of freezing time across the two‐tone presentations during the test session.

### Open Field Tests

A central area of 20 × 20 cm^2^ was defined in the field box (40 × 40 × 40 cm^3^). The mice were gently placed in the corner of the box and monitored for movement over a 10‐min period with an overhead camera in conjunction with the ANY‐maze tracking system (Stoelting, Wood Dale, IL, USA). The total distance traveled and time spent in the center of the box were recorded. After each mouse completed the trial, the box was cleaned with 70% ethanol.

### Foot‐Shock Sensitivity Test

The mice were transferred from their home cages to the fear conditioning chamber. Mice were then exposed to 0.5‐s foot shocks, delivered in 0.1‐mA increments starting at 0.1 mA, until three response thresholds were reached: noticing (orienting head movement), flinching (briefly raising the hind paws off the bars), and vocalizing. After each mouse completed the trial, the chamber was cleaned with 70% ethanol.

### Fiber Photometry

The mice were habituated to be connected to a fiber patch cord for 30 min over three consecutive days, followed by cue fear conditioning and memory retrieval as previously described. Fiber photometry experiments were performed with minor modifications^[^ ^61^ ^]^ via a fiber photometry system (Thinker Tech, Nanjing, China). In brief, fluorescent signals generated by a 488‐nm excitation laser beam were reflected by a dichroic mirror, focused through a 10× objective lens, and coupled to an optical commutator. The commutator and implanted fiber were connected via a 2‐m optical fiber (200 mm O.D., 0.37 NA). To minimize bleaching of the GCaMP6s probes, the laser power at the tip of the optical fiber was adjusted to 10–20 µW. Analog voltage signals were digitized at 100 Hz and recorded by the fiber photometry system. During fear memory retrieval, GCaMP6s fluorescence intensities were recorded, with the presound signal serving as the baseline.

The average calcium response was calculated via custom MATLAB codes (MATLAB R2017b, MathWorks). The data were segmented on the basis of behavioral events within individual trials. The fluorescence changes (ΔF/F) were calculated as (F − F0)/F0, where F0 represented the median fluorescence value during the baseline period (10 s preceding the onset of each CS). The ΔF/F values were displayed as heatmaps or per‐event plots, with shaded areas indicating the standard error of the mean (SEM). To statistically quantify changes in fluorescence values, the peak ΔF/F was defined as the maximal change during both the baseline (10‐s control window before CS onset) and the event (maximal values detected within 30 s after tone onset) periods during fear memory retrieval. Additionally, the area under the curve (ΔF/F×s) was calculated from the same set of data. To analyze the calcium dynamics during the event, the peak ΔF/F and area under the curve of the tone responses for the first 10 s of the CS were also quantified.

### TRAP Induction

In *TRAP2/Ai9* double transgenic mice, neuronal activation induces CreER expression, after 4‐hydroxytamoxifen (4‐OHT) injection, CreER translocates to the nucleus and triggers recombination.^[^ ^62^, ^63^ ^]^ This recombination results in the persistent expression of tdTomato (Cre reporter from Ai9) in active neurons. 4‐OHT (Sigma, Cat# H6278) was dissolved at 20 mg mL^−1^ in anhydrous ethanol by shaking at 37 °C for 15 min, aliquoted and stored at −20 °C for several weeks. The dissolved 4‐OHT was subsequently mixed with corn oil (Sigma, Cat #s259853) at a concentration of 10 mg mL^−1^ by shaking at 37 °C for 15 min. Afterward, ethanol was evaporated via vacuum centrifugation. The final 10 mg mL^−1^ 4‐OHT solution was injected intraperitoneally at a dose of 50 mg kg^−1^ 30 min after memory retrieval.^[^ ^62^ ^]^

### Stereotaxic Surgery

The mice were anesthetized with 1% sodium pentobarbital (100 mg kg^−1^, intraperitoneally) and placed in a stereotaxic frame (RWD Life Science Inc., China). At P21, viruses were injected into the PVT (anterior‐posterior: −1.20; medial‐lateral: 0.00; dorsal‐ventral: ‐3.25 mm relative to bregma) or CeA (anterior‐posterior: −1.15; medial‐lateral: ±2.80; dorsal‐ventral: −4.65 mm relative to bregma) of mice in the different groups at a flow rate of 20 nL min^−1^. After injection, the needles were left in place for 10 min before being withdrawn slowly to minimize viral particle leakage. Additional procedural details can be found in the literature.^[^ ^64^ ^]^

For fiber photometry, AAV‐EF1α‐DIO‐GCaMP6m (AAV2/9, 4.33E+13 vg mL^−1^, 150 nL; BrainVTA Co. Ltd., Wuhan, China) was injected into the PVT of *Vglut2*‐Cre mice. Optical fibers (200 µm O.D., 0.37 NA; Thinker Tech Nanjing Bioscience, Nanjing, China) were then implanted 50 µm above the viral injection coordinates in the PVT. For optogenetic manipulations, the PVT was injected with AAV‐EF1α‐DIO‐hChR2‐eYFP, AAV‐EF1α‐DIO‐eNpHR3.0‐eYFP, or AAV‐EF1α‐DIO‐eYFP (AAV2/9, 4.33E+13 vg mL^−1^, 150 nL each; BrainVTA Co. Ltd., Wuhan, China). Optical fibers (200 µm O.D., 0.37 NA; Thinker Tech Nanjing Bioscience, Nanjing, China) were then implanted into the PVT or bilateral CeA regions of *Vglut2*‐Cre mice 50 µm above the viral injection coordinates.

For anterograde tracing, AAV‐DIO‐eYFP (AAV2/9, 5.12×1012 vg mL^−1^, 150 nL; BrainVTA Co. Ltd., Wuhan, China) was injected into the PVT of *Vglut2*‐Cre mice. For retrograde tracing, AAV‐Retro‐DIO‐eYFP (AAV2/R, 5.73×1012 vg mL^−1^, 150 nL; BrainVTA Co. Ltd., Wuhan, China) was injected into the right CeA of *Vglut2*‐Cre mice. For the overexpression and knockdown experiments, AAV‐CMV‐DIO‐Igfbp2‐eYFP (AAV2/9, 5.00E+13 vg mL^−1^, 150 nL; BrainVTA Co. Ltd., Wuhan, China) or AAV‐CMV‐DIO‐(eYFP‐U6)‐Igfbp2 shRNA (AAV2/9, 5.00E+13 vg mL^−1^, 150 nL; BrainVTA Co. Ltd., Wuhan, China) was injected into the PVT region of *Vglut2‐*Cre mice. Additionally, AAV‐CMV‐DIO‐Igfbp2‐eYFP or AAV‐CMV‐DIO‐(eYFP‐U6)‐Igfbp2 shRNA was injected into the PVT, while AAV‐Retro‐Vglut2‐Cre was injected bilaterally into the CeA of wild‐type mice. AAV‐CMV‐DIO‐eYFP (AAV2/9, 5.00E+13 vg mL^−1^, 150 nL) and AAV‐U6‐CMV‐eYFP‐scramble shRNA (AAV2/9, 5.00E+13 vg mL^−1^, 150 nL) were used as controls.

### In Vivo Optogenetic Manipulation

The mice were connected to a laser diode (Thinker Tech Nanjing Bioscience, Nanjing, China) via an optical fiber cannula for 30 min over three consecutive days, followed by cue fear conditioning and memory retrieval experiments as previously described. The optical fiber was attached to a rotary joint (FRJ_1 × 1_FC‐FC, Doric Lenses, Canada), allowing free movement of the mice. Optogenetic experiments were performed with minor modifications by using an optogenetic system (Thinker Tech, Nanjing, China).^[^ ^64^ ^]^ For optogenetic activation, light pulses were synchronized with the fear conditioning system (XinRuan Informatics Co., China), delivering blue light (470 nm, 4–6 mW) in 5‐ms pulses at 10 Hz during fear memory retrieval. For optogenetic inhibition, yellow light (590 nm, 8–10 mW) was delivered continuously throughout fear memory retrieval experiments. In all experiments, light stimulation began 1 s before the sound and continued for 31 s, ensuring full coverage of the 30‐s CS exposure during fear memory retrieval.

### Whole‐Cell Recordings

Electrophysiological recording procedures followed the previous protocol.^[^ ^64^ ^]^ Mice were anesthetized with 1% sodium pentobarbital (100 mg kg^−1^, intraperitoneally) and perfused intracardially with 20 mL of ice‐cold oxygenated (95% O_2_ and 5% CO_2_) slice‐cutting solution containing (in mM): 93 N‐methyl‐D‐glucamine, 93 HCl, 2.5 KCl, 1.25 NaH_2_PO_2_, 10 MgSO_4_, 30 NaHCO_3_, 25 glucose, 20 HEPES, 5 sodium ascorbate, 3 sodium pyruvate, and 2 thiourea. The brain was removed after decapitation, and 300‐µm coronal sections were prepared using a vibratome (VT1200S; Leica, Germany). Slices were incubated in oxygenated artificial cerebrospinal fluid (aCSF) containing (in mM) 126 NaCl, 2.5 KCl, 1.25 NaH_2_PO_4_, 2 MgSO_4_, 10 glucose, 26 NaHCO_3_, and 2 CaCl_2_ and at 32 °C for at least 30 min, then equilibrated to room temperature (24–26 °C).

Whole‐cell patch‐clamp recordings were conducted on visually identified PVT neurons using a fluorescence microscope (BX51WI; Olympus, Japan) with a 40× water immersion lens and differential interference contrast optics. Recordings were performed at room temperature, with slices continuously superfused with oxygenated aCSF at 1–2 mL min^−1^. Patch electrodes (3–5 MΩ) were pulled from borosilicate glass capillaries via a pipette puller (P1000, Sutter Instrument, USA). The internal solution for recordings contained (in mM) 135 potassium gluconate, 5 NaCl, 10 HEPES, 1 EGTA, 0.3 Na‐GTP, 2 Mg‐ATP, and 1 MgCl_2_ (280‐300 mOsm, pH 7.2). Electrophysiological recordings were acquired with a MultiClamp 700B amplifier (Molecular Devices) and Clampex 10.6 software. Signals were low‐pass filtered at 2 kHz and digitized at 10 kHz with a Digidata 1440A (Axon Instruments, CA, USA). Experiments were discarded if the series resistance changed by more than 20%. APs were measured by injecting current pulses (500‐ms duration, 40‐ to 320‐pA intensity, with 40‐pA increments) in current‐clamp mode. Optical stimulation of ChR2‐ or NpHR‐expressing neurons was performed with a 200 µm optical fiber attached to a light‐emitting diode source (Thorlabs, USA). The efficacy of the ChR2‐expressing was confirmed by APs elicited by blue‐light stimulation (473 nm, 5 ms, and 10 Hz), whereas NpHR‐expressing was confirmed by the suppression of spiking during yellow‐light stimulation (590 nm, 500‐ms pulses).

### Bulk RNA Sequencing

PVT tissue samples were dissociated and homogenized for RNA isolation. Total RNA was extracted using TRIzol reagent (Ambion, Shanghai, China) per the manufacturer's instructions. RNA integrity was assessed with a Nanodrop ND‐2000 spectrophotometer (Thermo Fisher Scientific, Waltham, MA, USA), and samples with RNA integrity numbers above 7.0 were further analyzed using an Agilent 2100 Bioanalyzer (Agilent Technologies, Santa Clara, CA, USA). Library preparation was performed by Novogene Bioinformatics Technology Co., Ltd. (China) with a TruSeq RNA preparation kit (Illumina, San Diego, CA, USA) following manufacturer's protocols. Library quality was assessed with an advanced analytical fragment analyzer, and sequencing was performed on the Illumina HiSeq 6000 platform (Illumina, San Diego, CA, USA) to generate 125‐bp paired‐end reads. Raw RNA‐seq data were analyzed with FastQC (version 0.11.8; Babraham Bioinformatics, Babraham Institute, Cambridge, UK). Low‐quality reads were removed via Cutadapt v2.3 to ensure high‐quality reads for analysis. DEGs analysis was performed with the DESeq2 R package (1.16.1), adjusting *P* values using the Benjamini & Hochberg method. Significant differential expression was defined as an absolute fold change > 1 and *P* < 0.05. Raw sequence data were available in the Sequence Read Archive (SRA) at the National Center for Biotechnology Information (NCBI) under accession number PRJNA1178824.

### RT‐qPCR

Based on prior molecular data from our lab and established practices reported in previous studies, a sample size of n = 4 mice per group was selected for RNA‐seq validation.^[^ ^33^, ^65^, ^66^ ^]^ PVT tissue samples were dissociated and homogenized with a homogenizer for RNA isolation. Total RNA from the PVT was reverse transcribed to cDNA and amplified with a reverse transcription kit (Vazyme). The obtained cycle threshold (CT) values were then statistically analyzed. The relative mRNA expression of each target gene was quantified using GAPDH as the reference gene. The sequences of the primers (Sangon Biotech) used in this study are shown in Table S4 (Supporting Information).

### Western Blotting

PVT tissues were homogenized in RIPA lysis buffer containing a protease inhibitor cocktail. Protein concentration was measured via a bicinchoninic acid assay. Protein samples were separated on 10–15% SDS‐PAGE gels and transferred to a polyvinylidene fluoride membrane. Membranes were blocked at room temperature for 2 h and incubated with primary antibodies at 4 °C overnight. After incubation, membranes were washed with TBST (3 washes, 15 min each) and incubated with the secondary antibodies for 2 h. Protein bands were detected using enhanced chemiluminescence. After detection of each target protein, membranes were stripped, reblocked, and incubated with antibodies for additional target molecules.

Primary antibodies used included rabbit anti‐Igfbp2 (1:500, ab188200, Abcam), rabbit anti‐Lcn2 (1:500, ab216462, Abcam), rabbit anti‐Vwf (1:500, ab287962, Abcam), rabbit anti‐Hspa1b (1:500, ab5439, Abcam), and rabbit anti‐GAPDH (1:1000, AB‐P‐R001, GoodHere). The secondary antibody used was goat anti‐rabbit IgG (1:50,000, 111‐035‐003, Jackson ImmunoResearch Laboratories, Inc.). Protein expression of each target molecule was normalized to GAPDH expression.

### Immunofluorescence Staining

For immunofluorescence staining, brain sections were washed in PBS three times (10 min each), blocked at room temperature for 1 h, and then incubated with primary antibodies overnight at 4 °C. After incubation, sections were rinsed three times (10 min each) with PBS and incubated with a fluorescent dye‐conjugated secondary antibody for 1 h at room temperature. Following three additional PBS washes (10 min each), sections were incubated with DAPI.

Primary antibodies included mouse anti‐Igfbp2 (1:200, sc‐51534, Santa Cruz Biotechnology), rabbit anti‐GFAP (1:200, ab7260, Abcam), rabbit anti‐IBA1 (1:200, ab178847, Abcam), and rabbit anti‐NeuN (1:200, 24307, Cell Signaling Technology). The secondary antibodies included donkey anti‐rabbit Alexa Fluor 488 (1:500, Invitrogen) and donkey anti‐mouse Alexa Fluor 555 (1:500, Invitrogen). The fluorescence signals were visualized with a confocal microscope (Zeiss, LSM900).

### Golgi Staining

Golgi staining was performed with the FD Rapid Golgi Staining Kit (PK401, FD NeuroTechnologies, United States) according to the manufacturer's instructions. Brain samples were collected and immersed in a 1:1 mixture of solutions A and B in the dark at room temperature for 14 days. The samples were then transferred to solution C in the dark for 5 days, embedded in optimal cutting temperature compound, and frozen at −80 °C for 24 h. Coronal sections (100 µm) were cut using a cryostat microtome (Leica, Wetzlar, Germany). The staining procedure was completed following the manufacturer's instructions. Slides were examined under a confocal microscope with a 63× oil‐immersion objective lens (Zeiss, LSM900). Five basal dendrite segments, each 30 µm or longer, were randomly selected from each neuron. Dendritic spine density was analyzed with ImageJ.

### Statistical Analysis

Animals with incorrect virus microinjections or improper optic fiber placement were excluded from the analysis. Specifically, animals were excluded if viral expression or fiber placement deviated by more than ±200 µm from the intended target region, as verified by post hoc histology. All data were presented as the mean ± SEM. Data normality was assessed with the Shapiro‐Wilk test, and homogeneity of variance was determined with Bartlett test. Statistical comparisons were made via Student's t test or two‐way repeated‐measures analysis of variance (ANOVA), followed by Sidak's multiple comparisons. All statistical analyses were carried out with GraphPad Prism 10.0 (GraphPad Software) and *P* < 0.05 was considered statistically significant.

## Conflict of Interest

The authors declare no conflict of interest.

## Author Contributions

W.Z., K.P., and B.Z. contributed equally to this work. F‐H.J, S‐Y.S and H‐Y.L conception and design of research. W‐M.Z, B‐J.Z, Y‐C.W, Q‐Y.X, Y‐N.G, and R‐X.W performed experiments. W‐M.Z, K‐P, X‐W.M, G.W, H‐B.X, L.D, and X‐S.S analyzed data. W‐M.Z, K.P and S‐Y.S prepared figures. W‐M.Z, K.P, and B‐J.Z drafted the manuscript. F‐H.J, S‐Y.S, H‐Y.L, J.T and H.L edited and revised the manuscript. All the authors have read and approved the paper.

## Supporting information



Supporting Information

Supplemental Table 1

Supplemental Table 2

Supplemental Table 3

Supplemental Table 4

## Data Availability

The data that support the findings of this study are available in the supplementary material of this article.
