# Supplementary material for: Igfbp2 Downregulation in PVT‐CeA Glutamatergic Circuits Drives Neonatal Anesthesia‐Induced Fear Memory Deficits
Source: Adv Sci (Weinh). 2025 Aug 13;12(41):e05025. doi: 10.1002/advs.202505025 (PMC12591187; doi:10.1002/advs.202505025)
Supplement: Supplementary file 1 — Supporting Information [file ADVS-12-e05025-s002.docx]

Supporting Information

**Igfbp2 Downregulation in PVT-CeA Glutamatergic Circuits Drives Neonatal Anesthesia-Induced Fear Memory Deficits**

Weiming Zhao, Ke Peng, Baojian Zhao, Xiaowen Meng, Gang Wang, Hanbing Xu, Li Deng, Xisheng Shan, Yichan Wang, Qiya Xu, Yanan Gao, Ruixia Weng, Hong Liu, Jin Tao, Huayue Liu, Shaoyong Song, Fuhai Ji

Supplementary Information for

**Igfbp2 Downregulation in PVT-CeA Glutamatergic Circuits Drives Neonatal Anesthesia-Induced Fear Memory Deficits**

Weiming Zhao, Ke Peng, Baojian Zhao, Xiaowen Meng, Gang Wang, Hanbing Xu, Li Deng, Xisheng Shan, Yichan Wang, Qiya Xu, Yanan Gao, Ruixia Weng, Hong Liu, Jin Tao, Huayue Liu, Shaoyong Song, Fuhai Ji

This file includes:

Figure S1 to S14

Table S1 to S4, also see Excel files for Tables S1, S2, S3 and S4

Supplementary Information

**
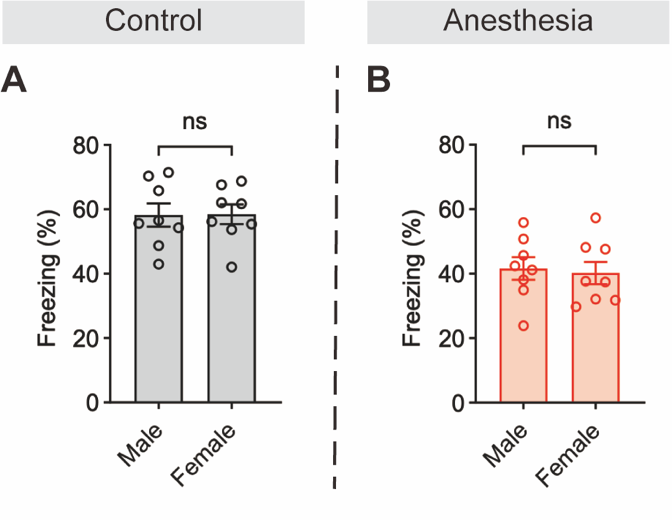
**

**Figure S1. Comparison between male and female mice revealed no differences in the extent of fear memory impairment induced by repeated neonatal anesthesia (related to Figure 1).**

No significant difference in fear memory retrieval between males and females in control (**A**) and anesthesia (**B**) groups; n = 8 mice per group. Analyzed by unpaired t test; ns: not significance. Data are presented as means ± SEM.

**
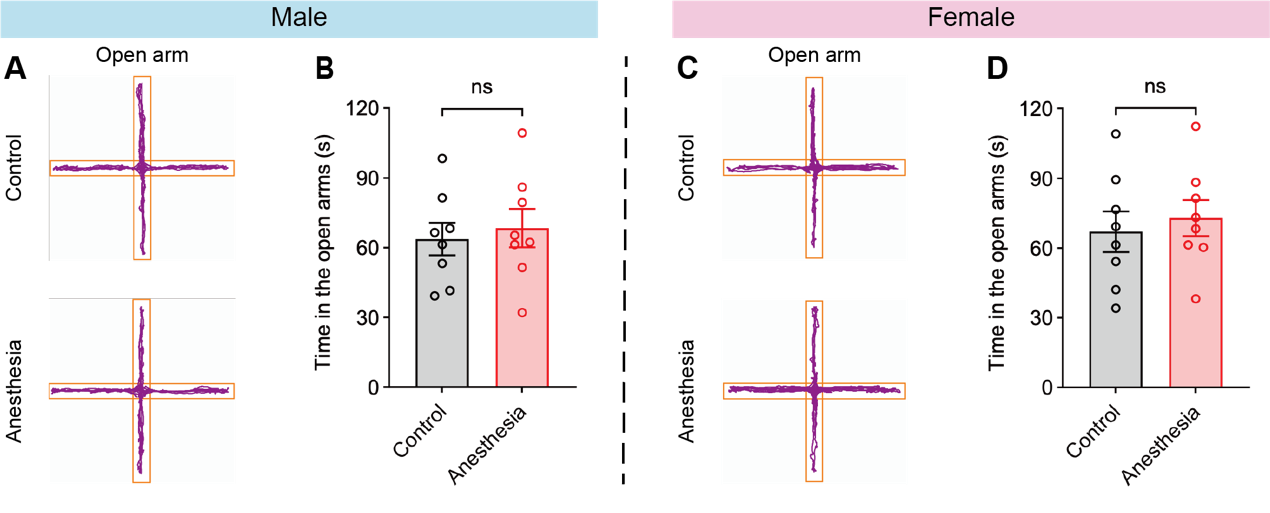
**

**Figure S2. Repeated neonatal anesthesia does not induce anxiety-like behavior in the elevated plus maze test (Related to Figure 1).**

**A, C** Representative trajectory plots of males (A) and females (C) mice during elevated plus maze testing.

**B, D** Time spent in the open arms showed no significant differences between control and anesthesia groups in either males (B) or females (D).

Analyzed by unpaired t test; n = 8 mice per group; ns: not significance. Data are presented as means ± SEM.

**
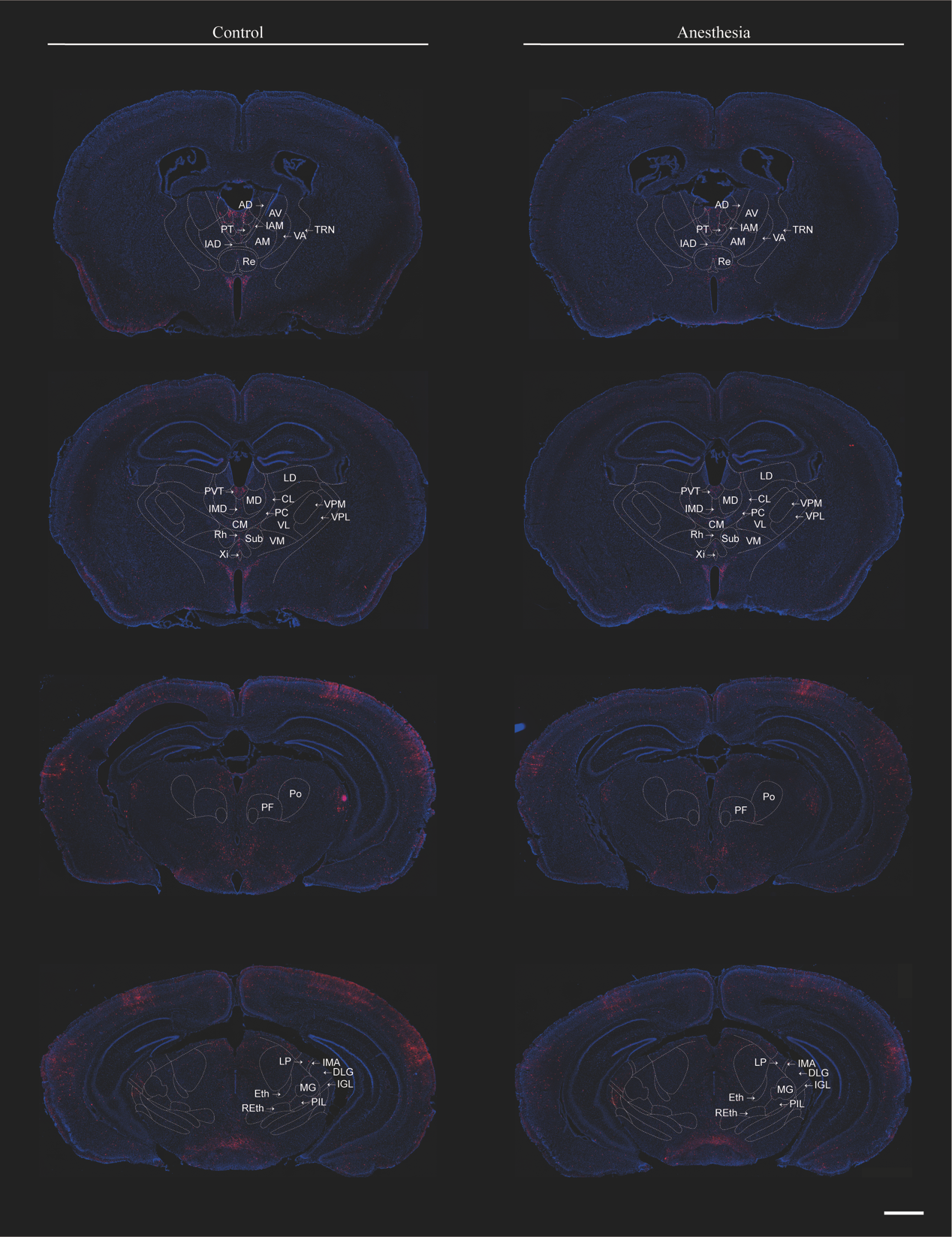
**

**Figure S3. Thalamus mapping of tdTomato^+^ expression in male *TRAP/Ai9* mice between control and anesthesia groups (related to Figure 2).**

Representative slices of the brain along the rostral-caudal axis showing tdTomato^+^ neurons in different areas and nuclei of thalamus; scale bar: 1 mm. AD: anterodorsal nucleus; AM: anteromedial nucleus; AV: anteroventral nucleus; IAD: interanterodorsal nucleus; IAM: interanteromedial nucleus; PT: paratenial nucleus; Re: reuniens nucleus; VA: ventral anterior nucleus; TRN: thalamic reticular nucleus; IMD: intermediodorsal nucleus; MD: mediodorsal nucleus; LD: laterodorsal nucleus; CL: centrolateral nucleus; CM: central medial nucleus; PC: paracentral nucleus; VL: ventral lateral nucleus; VM: ventromedial nucleus; PVT: paraventricular nucleus; Rh: rhomboid nucleus; Sub: submedius nucleus; Xi: xiphoid nucleus; VPM: ventral posteromedial nucleus; VPL: ventral posterolateral nucleus; Po: posterior nuclear group; PF: parafascicular nucleus; DLG: dorsal lateral geniculate nucleus; IMA: intramedullary thalamic area; MG: medial geniculate nucleus; IGL: intergeniculate leaflet; PIL: posterior intralaminar nucleus; Eth: ethmoid nucleus; REth: retroethmoid nucleus; LP: lateral posterior nucleus


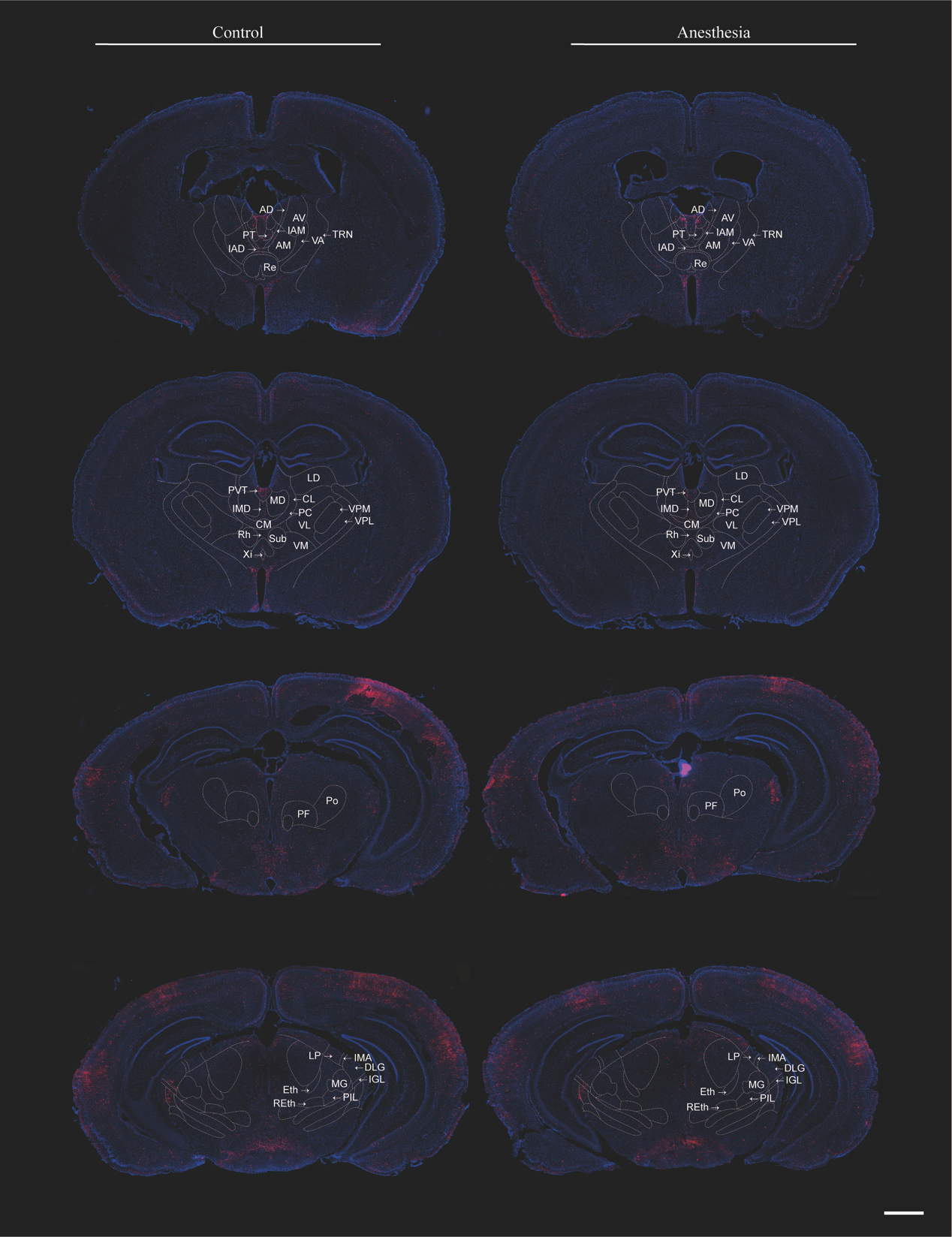


**Figure S4. Thalamus mapping of tdTomato^+^ expression in female *TRAP2/Ai9* mice between control and anesthesia groups (related to Figure 2).**

Conventions are the same as in Figure S3; scale bar: 1 mm.


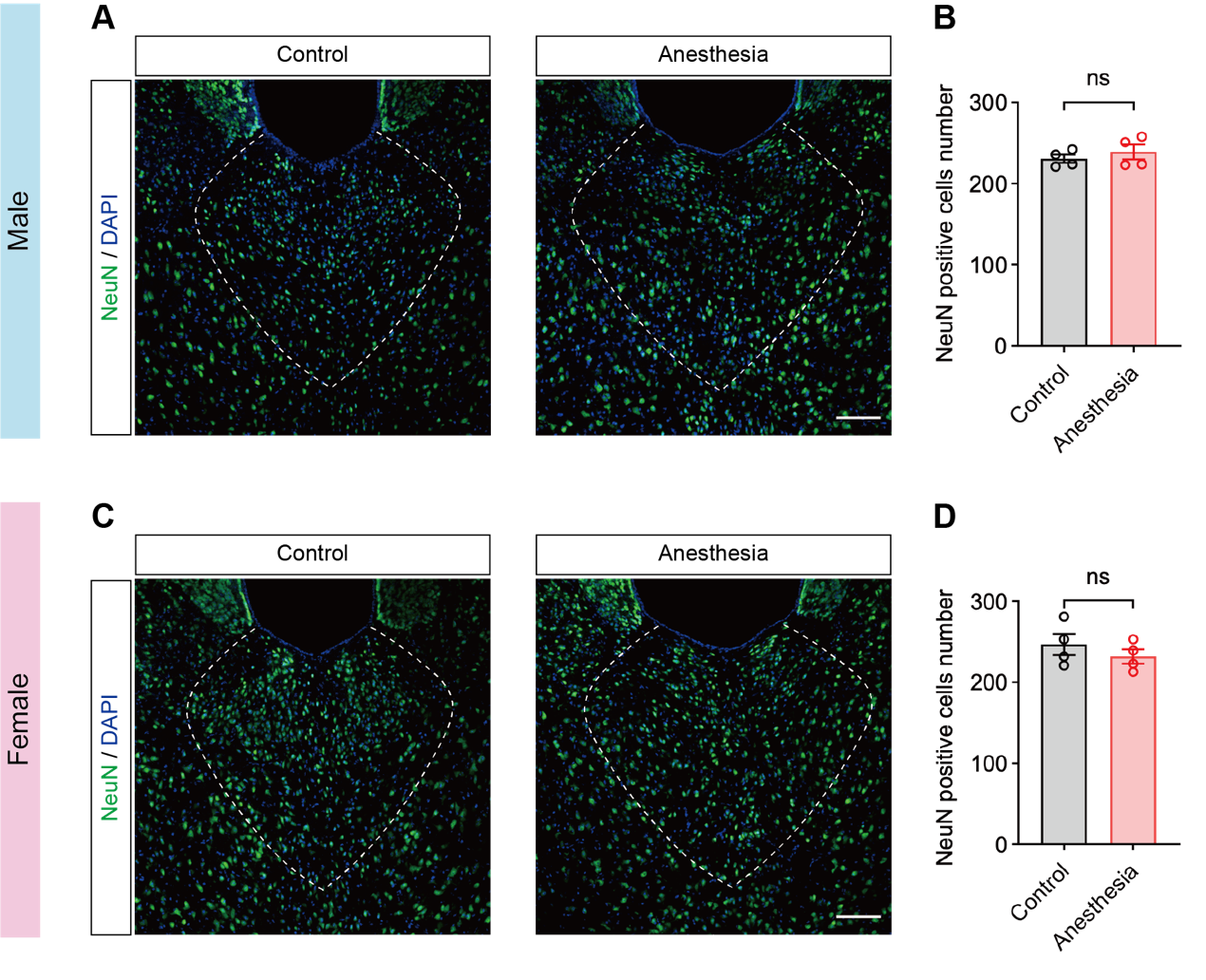


**Figure S5. Repeated neonatal anesthesia did not induce PVT neuronal loss in male or female adolescent mice (related to Figure 2).**

**A**, **B** Representative confocal images (**A**) and quantitative analysis of NeuN-positive cells in PVT (**B**) of males; scale bar: 100 μm.

**C**, **D** Representative confocal images (**C**) and quantitative analysis of NeuN-positive cells in PVT (**D**) of females, scale bar: 100 μm.

Analyzed by unpaired t test; n = 4 brain sections from 4 mice per group; ns: not significance. Data are presented as means ± SEM.


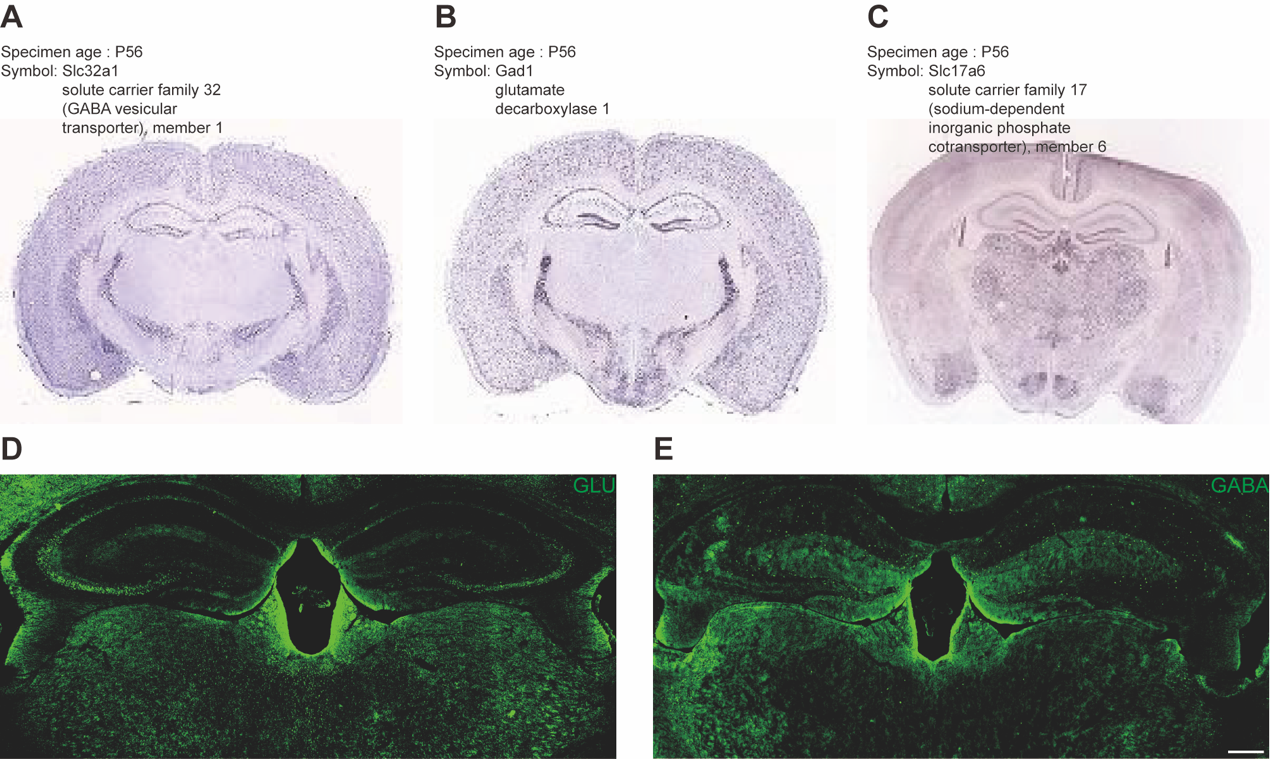


**Figure S6. Absence of GABAergic interneurons in PVT (related to Figure 3).**

**A**-**C** Representative in situ hybridization images from the Allen Brain Database showing the distribution of Slc32a1 (**A**), Gad1 (**B**) and Slc17a6 (**C**) mRNA in the PVT.

**D**-**E** Representative immunofluorescence images showing GLU^+^ (glutamatergic) neurons (**D**) and the absence of GABA^+^ (GABAergic) neurons (**E**) in the PVT; scale bar: 200 μm.


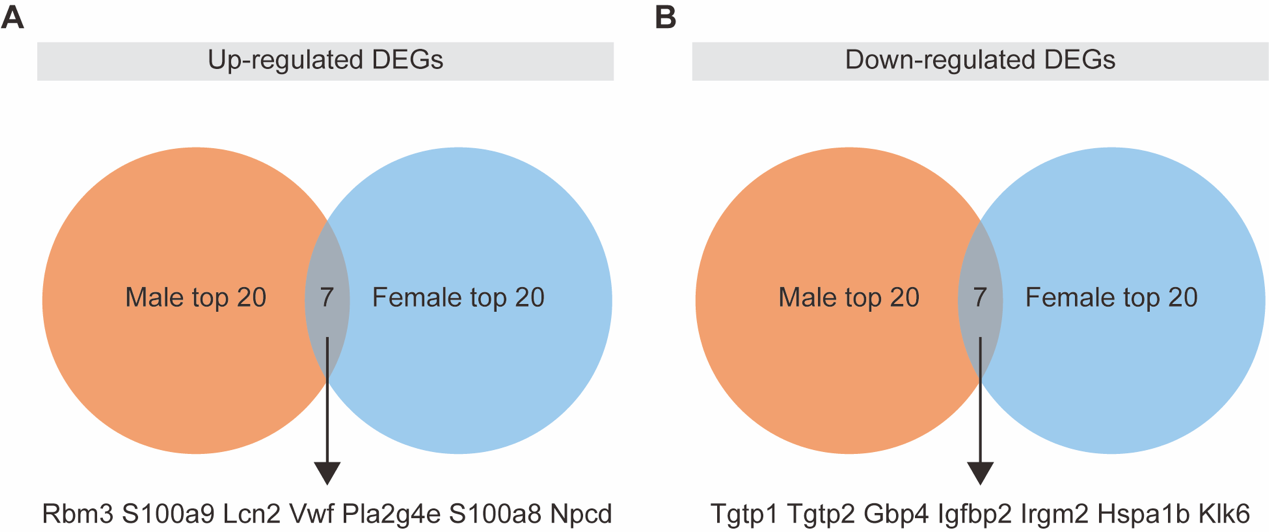


**Figure S7. Venn diagrams showing the overlap of top DEGs in PVT of male and female mice (related to Figure 5).**

**A** Venn diagram depicting the overlap of the top 20 up-regulated DEGs in males and females.

**B** Venn diagram depicting the overlap of the top 20 down-regulated DEGs in males and females.


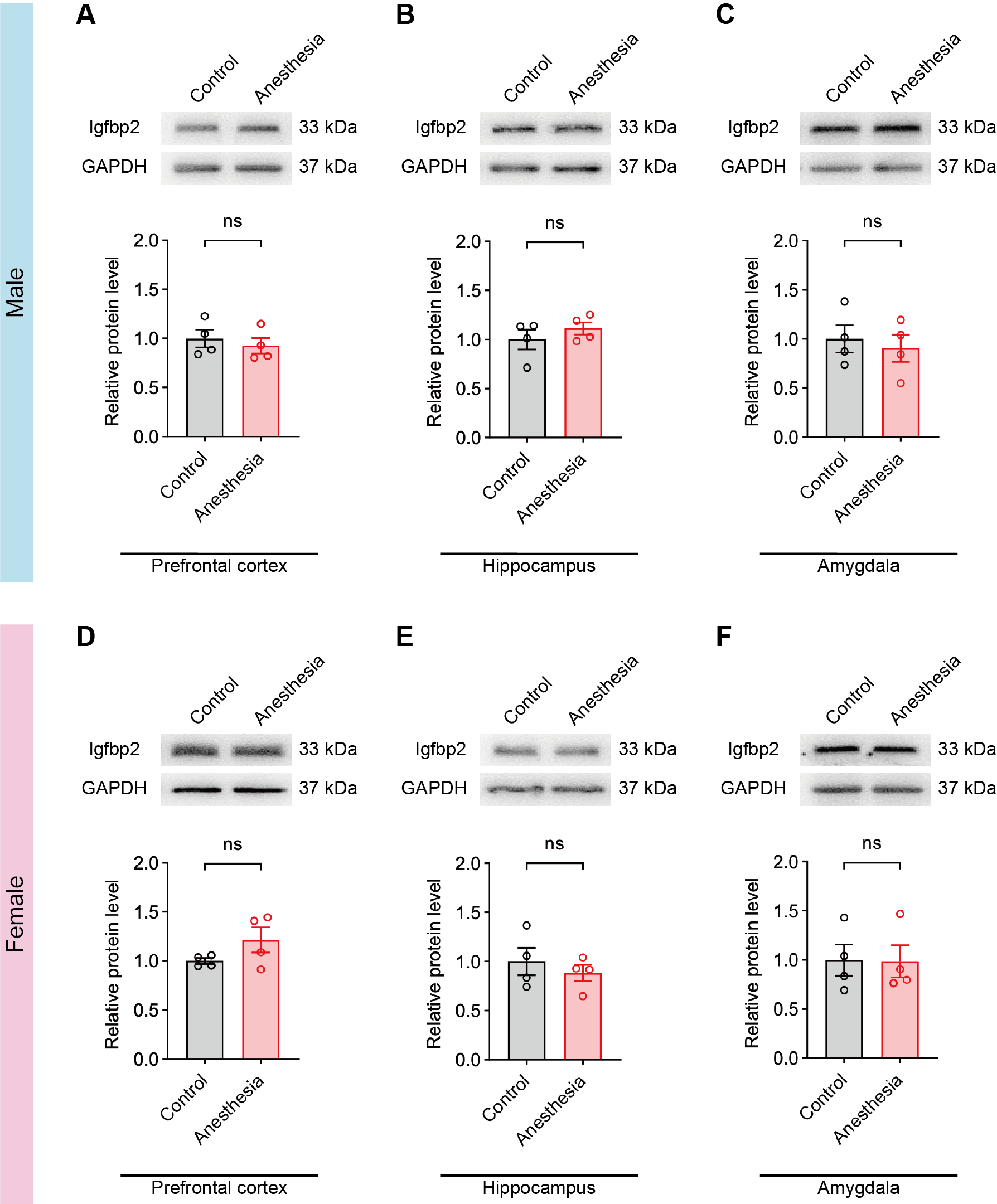


**Figure S8. Igfbp2 protein expression in different brain regions following repeated neonatal anesthesia (related to Figure 5).**

**A**-**C** Representative western blot bands (top) and quantification analysis (down) of Igfbp2 expression in prefrontal cortex (**A**) hippocampus (**B**) and amygdala (**C**) between control and anesthesia groups in males.

**D**-**F** Representative western blot bands (top) and quantification analysis (down) of Igfbp2 expression in prefrontal cortex (**D**) hippocampus (**E**) and amygdala (**F**) between control and anesthesia groups in females.

Analyzed by unpaired t test; n = 4 mice per group; ns: not significance. Data are presented as means ± SEM.


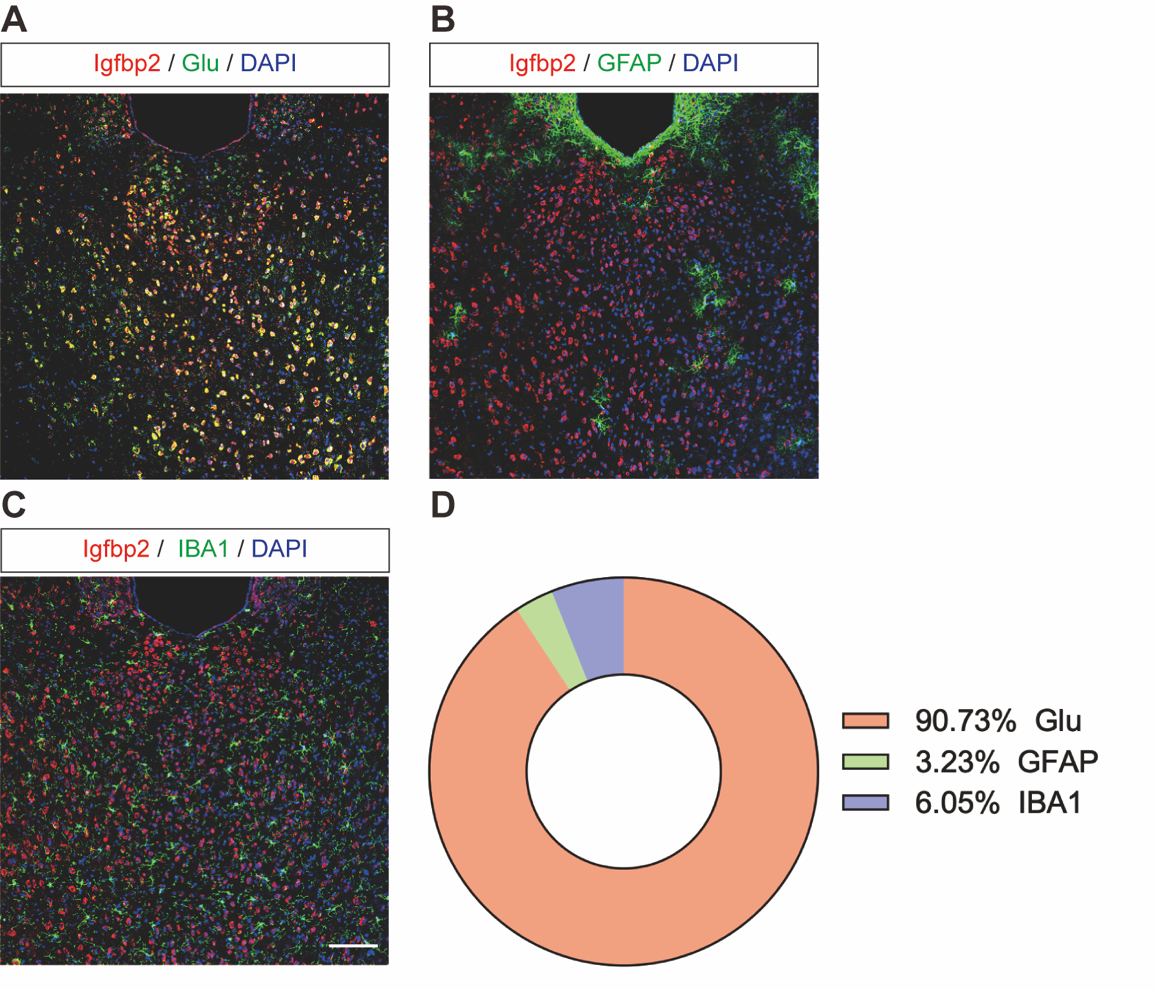


**Figure S9. Igfbp2 was predominantly expressed in PVT glutamatergic neurons (related to Figure 5).**

Representative images (**A**-**C**) and quantitative analysis (**D**) showing that Igfbp2 was highly co-expressed with Glu, with sparse co-expression with GFAP or IBA1; n = 4 brain sections from 4 mice per group; scale bar: 100 μm.


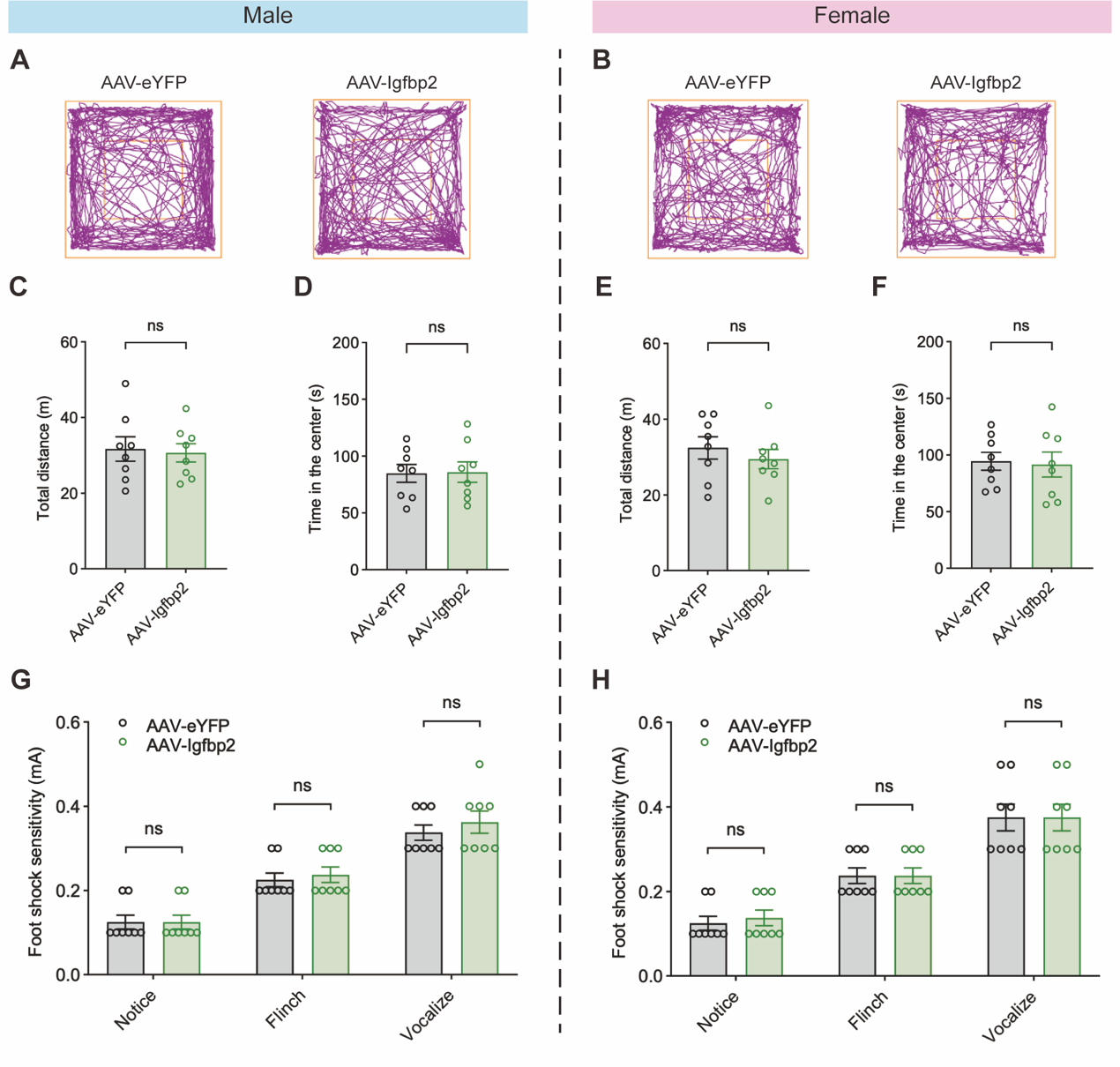


**Figure S10. Overexpression of Igfbp2 in PVT glutamatergic neurons did not induce locomotor dysfunction, anxiety-like behavior, or affect foot shock sensitivity (related to Figure 6).**

**A**, **B** Representative trajectory plots of males (**A**) and females (**B**) in open field test.

**C**, **E** Total distance traveled in the open field arena showed no significant differences between AAV-eYFP and AAV-Igfbp2 groups in males (**C**) and females (**E**).

**D**, **F** Time spent in the center region of the open field arena also showed no significant differences between AAV-eYFP and AAV-Igfbp2 groups in males (**D**) and females (**F**).

**G**, **H** No significant difference in foot shock sensitivity between AAV-eYFP and AAV-Igfbp2 groups in males (**G**) and females (**H**).

Analyzed by unpaired t test; n = 8 mice per group; ns: not significance. Data are presented as means ± SEM.

**
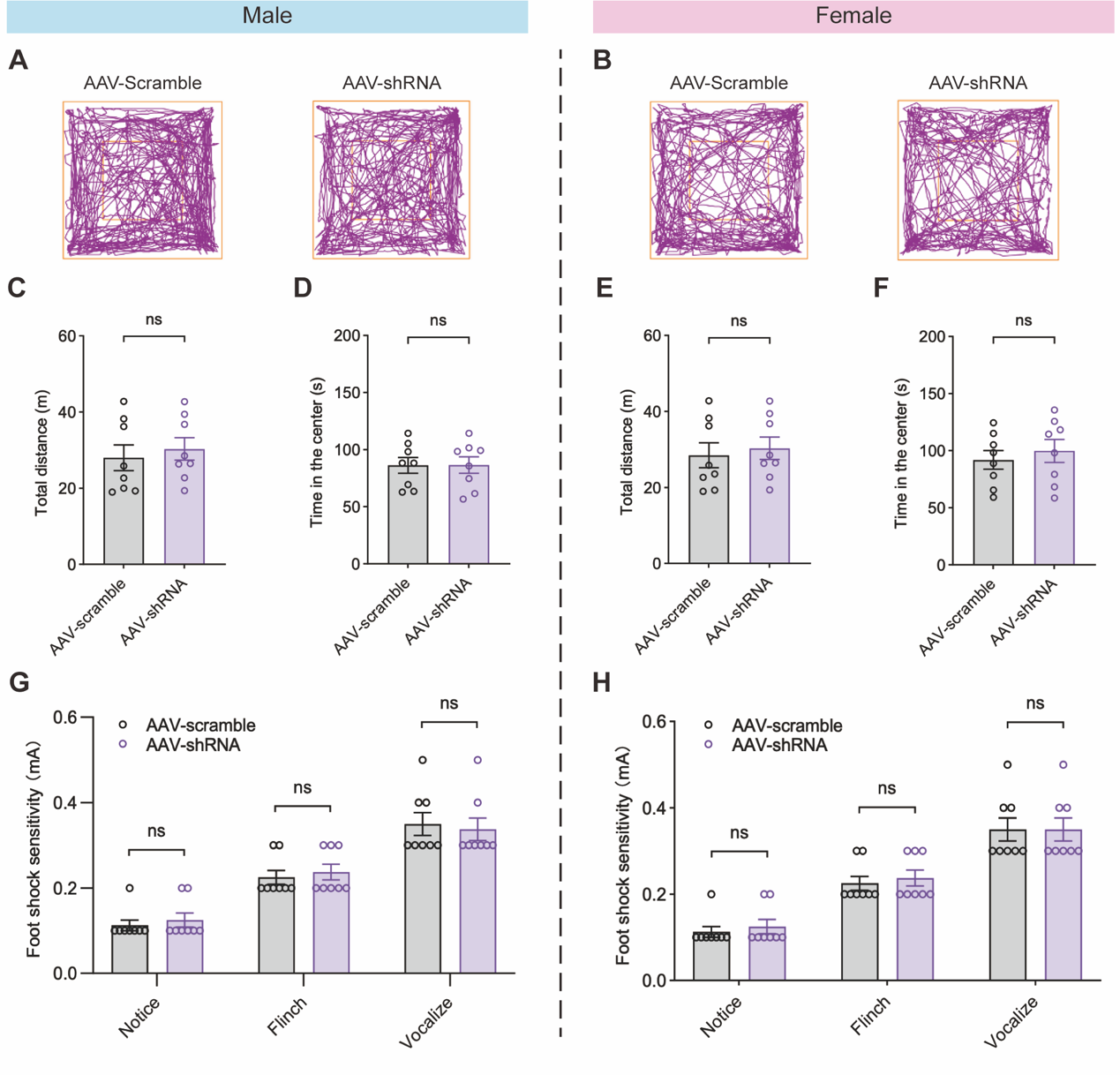
**

**Figure S11. Knockdown of Igfbp2 in PVT glutamatergic neurons did not induce locomotor dysfunction, anxiety-like behavior, or affect foot shock sensitivity (related to Figure 7).**

**A**, **B** Representative trajectory plots of males (**A**) and females (**B**) in open field test;

**C**, **E** Total distance traveled in the open field arena showed no significant difference in males (**C**) and females (**E**).

**D**, **F** Time spent in the center region of the open field arena also showed no significant difference in males (**D**) and females (**F**).

**G**, **H** No significant difference in foot shock sensitivity between AAV-scramble and AAV-shRNA groups in males (**G**) and females (**H**).

Analyzed by unpaired t test; n = 8 mice per group; ns: not significance. Data are presented as means ± SEM.


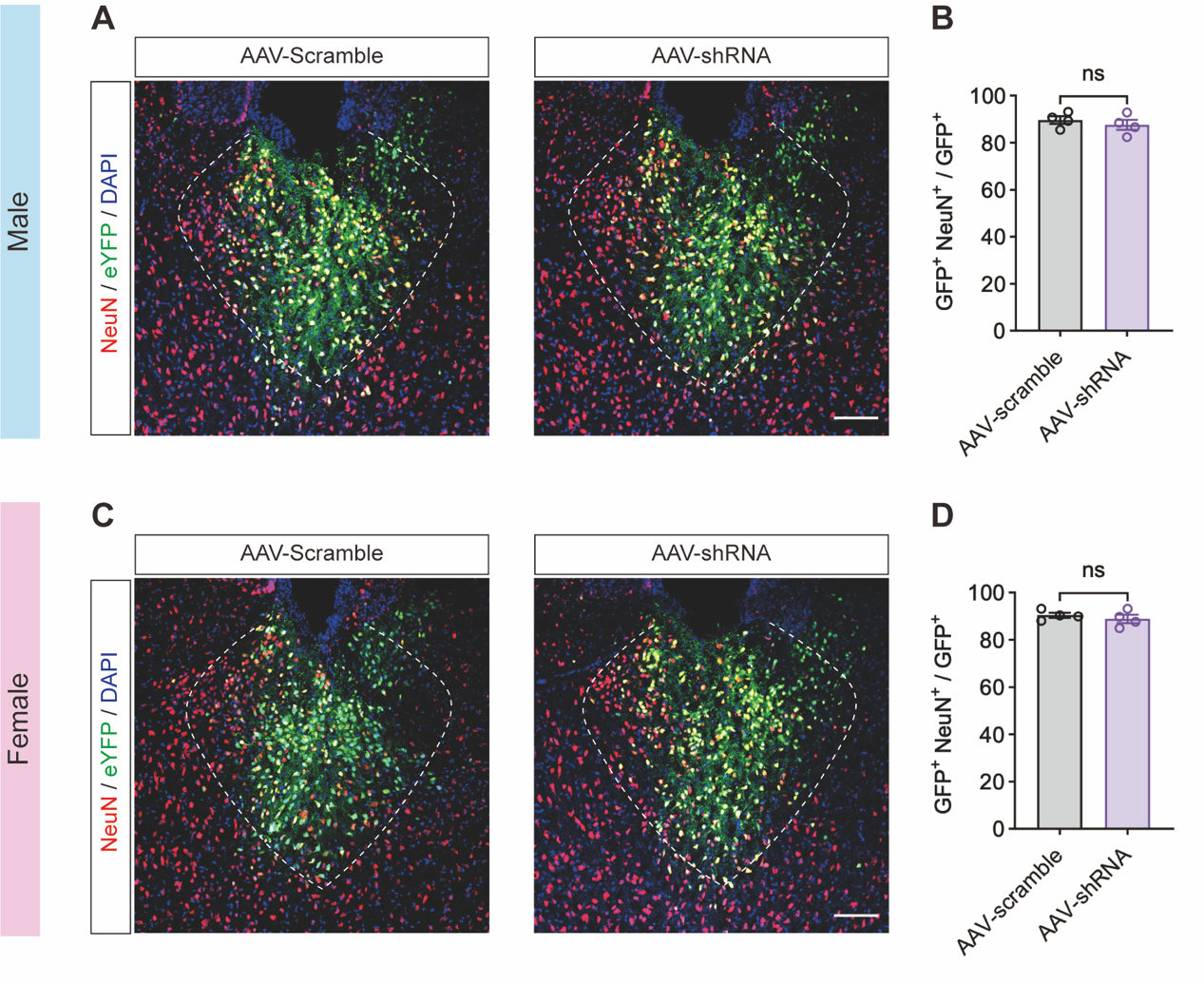


**Figure S12. Knockdown of Igfbp2 in PVT glutamatergic neurons did not affect the number of neurons (related to Figure 7).**

**A**, **B** Representative confocal images (**A**) and quantitative analysis of NeuN-positive cells in PVT (**B**) of males, scale bar: 100 μm.

**C**, **D** Representative confocal images (**C**) and quantitative analysis of NeuN-positive cells in PVT (**D**) of females, scale bar: 100 μm.

Analyzed by unpaired t test; n = 4 brain sections from 4 mice per group; ns: not significance. Data are presented as means ± SEM.


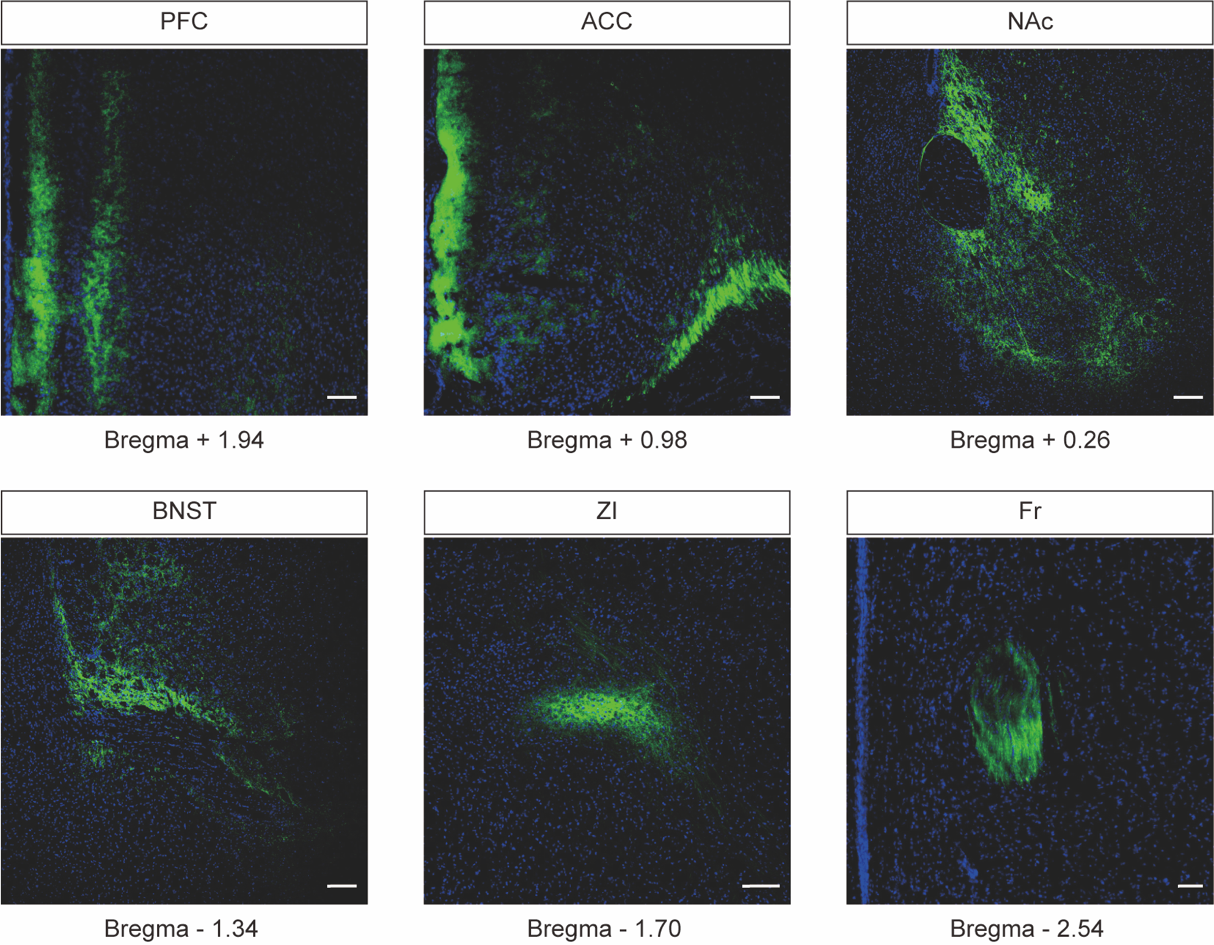


**Figure S13. Whole-brain mapping of direct outputs from vglut2^+^ neurons of PVT (related to Figure 8).**

Representative images showing projections at six coronal levels from vglut2^+^ neuron populations in PVT; scale bars: 100 μm. PFC, prefrontal cortex; ACC, anterior cingulate cortex; NAc, nucleus accumbens; BNST, bed nucleus of the stria terminalis; ZI, zona incerta; Fr, fasciculus retroflexus.


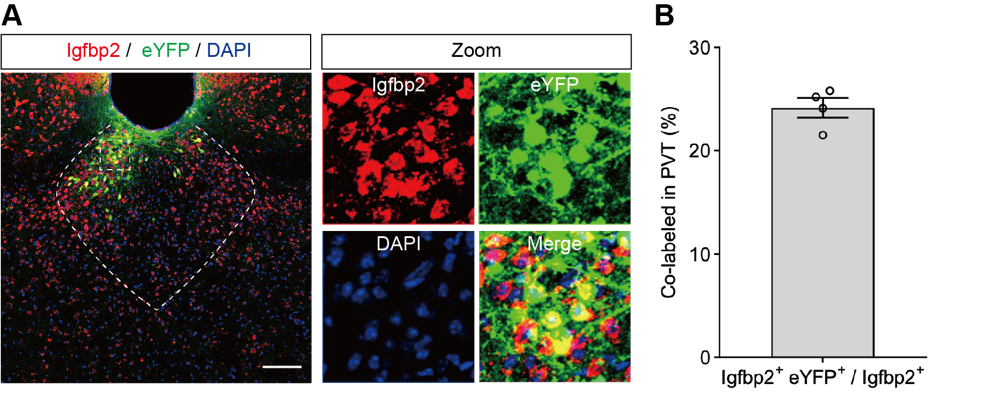


**Figure S14. Quantitative analysis of Igfbp2 expression in CeA-projecting PVT neurons (related to Figure 9).**

**A,** Representative immunofluorescence images showing retrograde labeling of CeA-projecting PVT neurons (eYFP, green) and Igfbp2 expression (red), with merged image demonstrating colocalization (yellow); scale bar: 100 μm.

**B,** Quantitative analysis of Igfbp2 expression in CeA-projecting PVT neurons; n = 4 brain sections from 4 mice per group. Data are presented as means ± SEM.

**Table S1. DEGs of male mice (related to Figure 5).**

Provided as a separate Excel file

**Table S2. DEGs of female mice (related to Figure 5).**

Provided as a separate Excel file

**Table S3. Top 20 up- and down-regulated DEGs for both sexes (related to Figure S6).**

Provided as a separate Excel file

**Table S4. Sequences of PCR primers (related to Figure 5).**

Provided as a separate Excel file
